# Health and Economic Impact of Periodic Hepatitis C Virus Testing Among People Who Inject Drugs

**DOI:** 10.1001/jamahealthforum.2025.1870

**Published:** 2025-07-03

**Authors:** Lin Zhu, Nathan W. Furukawa, William W. Thompson, Marissa B. Reitsma, Liisa M. Randall, Michelle Van Handel, Alice K. Asher, Eduardo Valverde, Benjamin P. Linas, Joshua A. Salomon

**Affiliations:** 1 Department of Health Policy, School of Medicine, Stanford University, Stanford, California; 2 National Center for HIV, Viral Hepatitis, STD, and TB Prevention, Centers for Disease Control and Prevention, Atlanta, Georgia; 3 Bureau of Infectious Disease and Laboratory Sciences, Massachusetts Department of Public Health, Boston; 4 Section of Infectious Disease, Department of Medicine, Boston Medical Center, Boston, Massachusetts; 5 Department of Epidemiology, Boston University School of Public Health, Boston, Massachusetts

## Abstract

**Importance:**

Periodic hepatitis C virus (HCV) testing is recommended for people who inject drugs (PWID), but the optimal testing frequency remains unknown.

**Objective:**

To evaluate the health benefits, costs, and cost-effectiveness of alternative HCV testing frequencies for PWID.

**Design, Setting, and Participants:**

This cost-effectiveness analysis extended a previously published agent-based network simulation model of HCV transmission through the sharing of injection equipment among PWID. Network-based HCV transmission was calibrated to longitudinal data from the Social Networks Among Appalachian People study and published literature on PWID networks in the US to evaluate HCV testing strategies in both a sparse PWID network setting with lower HCV transmission and a dense network setting with higher HCV transmission. Data were collected from November 2008 to August 2010, and data were analyzed from September 2017 to December 2019.

**Exposures:**

Periodic HCV testing and treatment, with alternative average testing frequencies among PWID who have access to and use HCV care.

**Main Outcomes and Measures:**

Changes in cumulative quality-adjusted life-years (QALYs) and health care costs over 60 years (in 2021 US dollars) and incremental cost-effectiveness ratios (ICERs) discounted at 3% annually.

**Results:**

The mean initial age of 1552 simulated PWID was 32 years. Compared with no testing, HCV testing and treatment among PWID over a 10-year intervention period increased QALYs by 2.5% to 4.6% and costs by 0.5% to 2.3% across average testing frequencies ranging from once every 2 years to once monthly. In a lower transmission setting, testing every 2 years was weakly dominated by more frequent testing strategies; testing every year, every 6 months, every 3 months, and every month had ICERs of $6000 per QALY, $9300 per QALY, $24 200 per QALY, and $138 400 per QALY, respectively. In a higher transmission setting, testing every 2 years and every year were both weakly dominated, while testing every 6 months, every 3 months, and every month had ICERs of $14 000 per QALY, $30 100 per QALY, and $93 300 per QALY, respectively. Results were sensitive to risks of primary infection and reinfection as well as access to and utilization of HCV testing services among PWID.

**Conclusions and Relevance:**

In this economic evaluation study, based on common benchmarks for cost-effectiveness, frequent HCV testing among PWID was cost-effective in both lower and higher transmission settings.

## Key Points

**Question:**

What are the health benefits, costs, and cost-effectiveness of periodic hepatitis C virus (HCV) testing among people who inject drugs (PWID)?

**Findings:**

In this economic evaluation study simulating 1552 PWID, using an agent-based network model over 60 years, HCV testing with average frequencies up to once monthly increased quality-adjusted life-years (QALYs) by up to 4.6% and costs by up to 2.3%, with incremental cost-effectiveness ratios from $6000 per QALY (every year) to $138 400 per QALY (every month).

**Meaning:**

Frequent testing among PWID may be effective and cost-effective as a component of plans to eliminate hepatitis C.

## Teaser

This economic evaluation study evaluates the health benefits, costs, and cost-effectiveness of alternative hepatitis C virus testing frequencies for people who inject drugs.

## Introduction

The advent of direct-acting antiviral (DAA) treatments for hepatitis C virus (HCV) raised prospects for hepatitis C elimination because the treatments are highly efficacious, have mild adverse effects, and require only 8 to 12 weeks of oral medication.^1^ The US has adopted the World Health Organization’s goal of hepatitis C elimination by 2030.^2,3^ However, due in part to the asymptomatic nature of HCV infection, approximately one-third of people living with chronic hepatitis C in the US are unaware of their infection.^4^ In 2020, the US Centers for Disease Control and Prevention^5^ and the US Preventive Services Task Force^6^ updated their HCV screening guidelines to recommend screening at least once in a lifetime for all adults and periodic testing among certain individuals, including people who inject drugs (PWID), who account for most current and new HCV infections in the US. However, the optimal interval of HCV testing among PWID is unknown.

More testing and treatment may lead to greater reductions in hepatitis C cases; however, information on long-term health and economic impacts are needed to help policymakers understand and weigh potential benefits and costs of different strategies, given DAA treatments cost approximately $19 000 to $25 000 per course in the US. Globally, studies have evaluated the cost-effectiveness of DAA treatment in PWID,^7,8,9^ different HCV testing techniques,^10,11,12,13^ and programs to improve HCV care.^14,15,16,17,18,19,20^ However, the cost-effectiveness of different testing frequencies among PWID remains unknown.

Among PWID, HCV is primarily transmitted through sharing of injection equipment (needles, syringes, and others). One challenge to estimating impact and cost-effectiveness of HCV testing and treatment among PWID is estimating the indirect benefits of hepatitis C cure through preventing future transmission, otherwise known as treatment as prevention.^21^ Agent-based network models simulate heterogeneous individual characteristics and partnerships between individuals, so that when one individual’s infection status changes, the impact on this individual’s partners can be explicitly modeled. In addition, PWID network characteristics and intensity of HCV transmission can vary between settings, which can affect intervention outcomes.^22^ In this study, we therefore use a dynamic, agent-based network model to simulate HCV transmission in 2 representative PWID network settings to evaluate the cost-effectiveness of different HCV testing frequencies.

## Methods

### Analytic Overview

**Figure 1.**
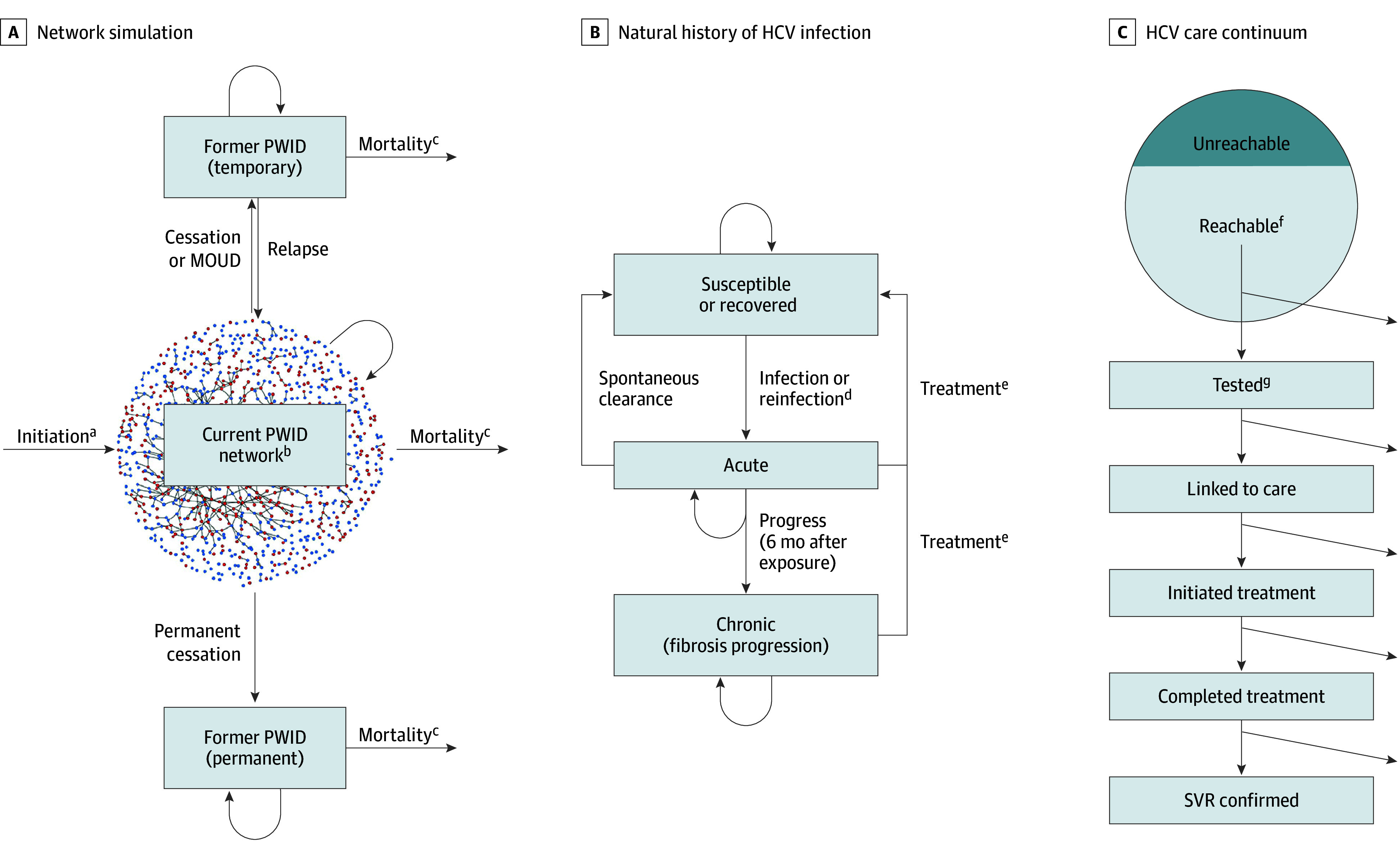
Network Simulation, Natural History of Hepatitis C Virus (HCV) Infection, and HCV Care Continuum C, The arrows out indicate attrition (those who are not tested or linked to care). MOUD indicates medications for opioid use disorder; PWID, people who inject drugs; SVR, sustained virologic response. ^a^Individuals who newly initiate injection drug use were assumed to have never been infected with HCV.^23^ ^b^HCV is transmitted via the injection network; if an individual is engaged in syringe services program, the probability of HCV transmission and acquisition is reduced. ^c^Mortality is dependent on each individual’s age, injection drug use status, HCV infection status, and fibrosis stage. ^d^Risk of reinfection during and after HCV treatment is reduced compared with risk of primary infection in the main analysis. ^e^Individuals who are receiving HCV treatment are assumed to be not infectious; individuals are eligible to be treated again if they are reinfected. ^f^Whether an individual can be reached by testing and treatment is determined by the coverage parameter and fixed. ^g^Monthly testing probability for each individual is determined by the testing frequency evaluated; individuals who test antibody positive will receive an RNA test automatically.

We developed a dynamic network simulation model of HCV transmission via injection equipment sharing among PWID. We incorporated injection behaviors, natural history of HCV infection, progression of liver conditions from untreated HCV infection, and the HCV testing and treatment cascade as well as harm-reduction interventions. We compared cumulative health outcomes and costs across different testing frequencies in the simulated networks. We conducted sensitivity analyses on key parameters and assumptions. A summary of the model is presented in Figure 1,^23^ and all model parameters are summarized in the Table.^24,25,26,27,28,29,30,31,32,33,34,35,36,37,38,39,40,41,42,43,44,45,46,47,48,49,50,51,52,53,54,55,56,57,58,59,60,61,62,63,64,65,66,67,68,69,70,71^ This study was determined by the Stanford University Institutional Review Board to be non–human subjects research. We used the reporting guidelines set forth by the Second Panel on Cost-Effectiveness in Health and Medicine^72^ for our study.

**Table.** Input Parameters for Simulations Using the Dynamic Network Model of People Who Inject Drugs (PWID)

Parameter	Value	Source
Injection drug use dynamics		
Monthly initiation rate^a^	0.002	Calibration
Monthly cessation rate^b^	0.014	Shah et al,^24^ 2006
Monthly relapse rate^b^	0.033	Shah et al,^24^ 2006
Probability of permanent cessation^b^	0.13	Shah et al,^24^ 2006
Mortality		
SMR for former PWID^c^	1.8	Evans et al,^25^ 2015
SMR for current PWID^c^	6.1	Evans et al,^25^ 2015
Additional monthly mortality rate for fibrosis stage 4^d^	0.0001	Bruno et al,^26^ 2009
Additional monthly mortality rate for decompensated^d^	0.0128	Bruno et al,^26^ 2009
Post-SVR mortality multiplier for fibrosis stage 4 and decompensated^d^	0.29	van der Meer et al,^27^ 2012
Network simulations		
Initial current PWIDs, No.	1000	Zhu et al,^28^ 2024; assumption
Initial former PWIDs, No.	373	SNAP; assumption
Baseline HCV antibody positivity, %	59	Zhu et al,^28^ 2024; SNAP
Mean degree in sparse network	1.43	Zhu et al,^28^ 2024; SNAP
Mean degree in dense network	3	Zhu et al,^28^ 2024; eTable 1 in Supplement 1
Ratio of mean degree between HCV antibody-positive and antibody-negative PWIDs	1.73	Zhu et al,^28^ 2024; SNAP
Proportion of population with no partners (isolates), %	37	Zhu et al,^28^ 2024; SNAP
HCV serodiscordant partnerships, %	29	Zhu et al,^28^ 2024; SNAP
Transitivity (GWESP density)^e^	0.28	Zhu et al,^28^ 2024; SNAP
Average equipment-sharing partnership duration, y^f^	3	Morris et al,^29^ 2014; SNAP; assumption
HCV infection and progression		
Monthly transmission probability in discordant partnerships	0.031	Zhu et al,^28^ 2024 (calibration)
Spontaneous clearance of acute infection, %	25	Smith et al,^30^ 2016
Probability of HCV seroconversion after different period of time following acute infection^g^		
1 mo	0.05	Busch and Page Shafer,^31^ 2005; Kim and Corcorran,^32^ 2024; Stramer et al,^33^ 2004
2 mo	0.5	
3 mo	0.9	
≥4 mo	0.99	
Monthly probability of progression from fibrosis stage 0 to 1	0.008877	Erman et al,^34^ 2019
Monthly probability of progression from fibrosis stage 1 to 2	0.00681	
Monthly probability of progression from fibrosis stage 2 to 3	0.0097026	
Monthly probability of progression from fibrosis stage 3 to 4	0.0096201	
Monthly probability of progression from fibrosis stage 4 to decompensation	0.0097026	
HCV testing and DAA treatment		
Testing coverage, %^h^	60	Smith et al,^35^ 2020; Barocas et al,^36^ 2014; Hajarizadeh et al,^37^ 2023
HCV antibody test specificity^i^	0.99945	US Food and Drug Administration,^38^ 2025
HCV antibody test sensitivity^i^	1.00	US Food and Drug Administration,^38^ 2025
HCV RNA test specificity^i^	0.999985	US Food and Drug Administration,^38^ 2025
HCV RNA testing sensitivity^i^	1.00	US Food and Drug Administration,^38^ 2025
Linkage to care (ie, scheduled follow-up clinician appointment), %	46.9	Viner et al,^39^ 2015; Falade-Nwulia et al,^40^ 2020; Seña et al,^41^ 2016
Average interval between positive test result and treatment initiation, mo	3	Harris et al,^42^ 2021
Treatment initiation among those linked to care, %	92	Younossi et al,^43^ 2016
Treatment completion among those who initiate, %	96	Berg et al,^44^ 2019; Cornberg et al,^45^ 2022; Sowah et al,^46^ 2023; Hajarizadeh et al,^47^ 2018
Cure rate (SVR among those who complete treatment), %	94	Hajarizadeh et al,^47^ 2018; Cunningham et al,^48^ 2018
RNA testing rate after treatment completion, %^j^	76	Cornberg et al,^45^ 2022
Relative risk of reinfection after cure (compared with primary infection)^k^	0.34	Hajarizadeh et al,^49^ 2020; Esmaeli et al,^50^ 2017; Valencia et al,^51^ 2019; Caven et al,^52^ 2019
Effects of SSP and MOUD		
Relative reduction in transmission probability when engaged in SSP, %	50	Platt et al,^53^ 2018
Engagement duration for SSP, mo	12	Gindi et al,^54^ 2009
Engagement duration for MOUD, mo	4	Krawczyk et al,^55^ 2021
Health utility		
Base (age 14-29 y)	0.928	Hanmer et al,^56^ 2006
Base (age 30-39 y)	0.918	Hanmer et al,^56^ 2006
Base (age 40-49 y)	0.887	Hanmer et al,^56^ 2006
Base (age 50-59 y)	0.861	Hanmer et al,^56^ 2006
Base (age 60-69 y)	0.840	Hanmer et al,^56^ 2006
Base (age 70-79 y)	0.802	Hanmer et al,^56^ 2006
Base (age 80-100 y)	0.782	Hanmer et al,^56^ 2006
Current PWID	0.68	Pyne et al,^57^ 2008
Former PWID	0.82	Pyne et al,^57^ 2008
Fibrosis stage 0 to 3, with HCV infection^l^	0.96	van der Meer et al,^27^ 2012; Saeed et al,^58^ 2020
Fibrosis stage 4, with HCV infection^l^	0.85	van der Meer et al,^27^ 2012; Saeed et al,^58^ 2020
Decompensated, with HCV infection^l^	0.77	van der Meer et al,^27^ 2012; Saeed et al,^58^ 2020
Fibrosis stage 0 to 3, SVR or no infection^l^	1	van der Meer et al,^27^ 2012; Saeed et al,^58^ 2020
Fibrosis stage 4, SVR or no infection^l^	0.96	van der Meer et al,^27^ 2012; Saeed et al,^58^ 2020
Decompensated, SVR or no infection^l^	0.93	van der Meer et al,^27^ 2012; Saeed et al,^58^ 2020
Costs, 2021 US dollars		
Health care excluding HCV testing and treatment (monthly)		
Current PWID^m^	2014	McCollister et al,^59^ 2018; Murphy et al,^60^ 2019; Lee et al,^61^ 2016
Former PWID^m^	1486	McCollister et al,^59^ 2018; Murphy et al,^60^ 2019; Lee et al,^61^ 2016
PWID engaging in MOUD^m^	1075	McCollister et al,^59^ 2018; Murphy et al,^60^ 2019; Lee et al,^61^ 2016
Fibrosis stage 0 to 4, active infection^n^	310	Chhatwal et al,^62^ 2013; McAdam-Marx et al,^63^ 2011
Fibrosis stage 0 to 4, SVR or no infection^n^	155	Chhatwal et al,^62^ 2013; McAdam-Marx et al,^63^ 2011; Davis et al,^64^ 2011
Decompensated, active infection^n^	2396	Chhatwal et al,^62^ 2013; McAdam-Marx et al,^63^ 2011
Decompensated, SVR or no infection^n^	1198	Chhatwal et al,^62^ 2013; McAdam-Marx et al,^63^ 2011; Davis et al,^64^ 2011
HCV testing		
Antibody test^o^	14.27	US Centers for Medicare & Medicaid Services,^65^ 2024
RNA test^o^	35.09	US Centers for Medicare & Medicaid Services,^65^ 2024
Personnel (HCV RNA positive)^p^	71	Schackman et al,^66^ 2018
Personnel (HCV RNA negative)^p^	67	Schackman et al,^66^ 2018
Linkage to care^p^	94	Schackman et al,^66^ 2018
Treatment initiation^q^	NA	American Association for the Study of Liver Diseases,^67^ 2023
New clinician visit (80% level 4 or 20% level 3)	142.78	US Centers for Medicare & Medicaid Services,^68^ 2024
CBC	6.47	US Centers for Medicare & Medicaid Services,^65^ 2024
Liver function tests	8.17	US Centers for Medicare & Medicaid Services,^65^ 2024
eGFR	5.18	US Centers for Medicare & Medicaid Services,^65^ 2024
HBV surface antigen testing	10.33	US Centers for Medicare & Medicaid Services,^65^ 2024
HIV antibody/antigen testing	24.08	US Centers for Medicare & Medicaid Services,^65^ 2024
DAA treatment (whole course of glecaprevir/pibrentasvir)	19 368.84	Office of Procurement Acquisition and Logistics,^69^ 2025
Treatment completion	NA	American Association for the Study of Liver Diseases,^67^ 2023
RNA test	35.09	US Centers for Medicare & Medicaid Services,^65^ 2024
Physician visit (level 2)	61.94	NA
Harm-reduction interventions (per client, monthly)		
SSP	100	Teshale et al,^70^ 2019
MOUD	550	Fairley et al,^71^ 2021

Abbreviations: CBC, complete blood count; DAA, direct-acting antiviral; eGFR, estimated glomerular filtration rate; GWESP, geometrically weighted edgewise shared partnerships; HBV, hepatitis B virus; HCV, hepatitis C virus; MOUD, medication for opioid use disorder; NA, not applicable; SMR, standardized mortality ratio; SNAP, Social Networks Among Appalachian People; SSP, syringe services program; SVR, sustained virologic response.

^a^ Number of new PWIDs per month was calculated by multiplying this rate and initial population size (n = 1000). Initiation rate was calibrated to keep population size of current PWID stable.

^b^ We used Kaplan-Meier estimators of time to cessation and relapse (given cessation) in the ALIVE study to estimate the 3 parameters (eAppendix in Supplement 1).

^c^ Overall SMRs for all ages; we generated age-dependent SMRs by fitting a regression to multiple SMR and age group points described in the eAppendix in Supplement 1.

^d^ Calculation is described in detail in the eAppendix in Supplement 1.

^e^ Proportion of 2 stars (2 nodes connected to a common node) that are closed (formed triangle), which describes the tendency of the phenomenon “a friend’s friend is more likely to be a friend.”

^f^ Analysis from the SNAP data shows average duration of acquaintance between injection partners was 10 years; in the cited study,^29^ the reported median duration of acquaintance was 10 months and duration of an injection partnership was 4.5 months. We hence assumed an average duration of 3 years in the main analysis.

^g^ Antibodies to HCV typically become detectable within 20 to 150 days after infection (mean, 60 days), ^31^ so we assumed 5% becoming detectable within 1 month after infection and 50% within 2 months after infection. After 12 weeks, more than 90% of patients will have a positive HCV antibody test result, ^32^ so we assumed 90% within 3 months after infection. In rare cases, there are individuals who do not develop antibody, and we assumed this percentage to be 1%. ^33^

^h^ Literature of urban PWID reported 80%. We assumed that the coverage in rural PWID is lower and hence added sensitivity analysis of 30% and 90%, spanning beyond 80%.

^i^ Using mean value of the approved assays.

^j^ We used percentage of people who use drugs that had SVR data to approximate this parameter; the cited study^45^ reported 87 people who use drugs, among whom 66 had SVR data.

^k^ In the 2 cited meta-analyses,^49,50^ the rate of HCV reinfection after treatment was 6.2 per 100 person-years among people who recently injected drugs, and the gender-weighted rate of HCV primary infection among PWID was 18 per 100 person-years. The relative risk of reinfection after treatment was thus estimated to be 0.34. Valencia et al^51^ reported high rates of early HCV reinfection after treatment and that 80% of people continued using drugs during treatment; and Caven et al^52^ reported that treatment reduced drug use but reported no change in sharing behavior. Based on these references, we used this same relative risk for reinfection during treatment.

^l^ Details of calculation are described in the eAppendix in Supplement 1.

^m^ The values presented are the means; we used age-dependent values specified in the eAppendix in Supplement 1. The values were obtained through communication with the authors (eAppendix in Supplement 1).

^n^ Details of calculation are described in the eAppendix in Supplement 1.

^o^ We simulated HCV reflex testing, in which people who test as antibody positive automatically get an RNA test.

^p^ We used the sum of pretest and posttest counseling costs to represent personnel cost and care coordination services to represent linkage cost^66^; for HCV-positive individuals, we used the mean of costs of patients with HIV/HCV coinfection and patients with HCV infection.

^q^ For DAA treatment initiation, we included components specified in HCV treatment guidance by the American Association for the Study of Liver Diseases.^67^ We assumed everyone who initiates DAA treatment would receive a whole course (8 weeks) of glecaprevir/pibrentasvir, which is a simplification of the real-world variation in use of DAA treatments.

### Network Simulation

Previously, we analyzed data from the Social Networks Among Appalachian People (SNAP) study, a longitudinal study of people who use drugs that collected information on demographic characteristics, drug use, and dyad-level characteristics of injection partnerships,^73,74,75,76^ as well as published literature on PWID in the US (eTable 1 in Supplement 1) to inform model inputs and calibration.^28,77^ We included age, HCV infection status, fibrosis stage, and injection drug use status (current vs former) for each individual. We simulated dynamic injection equipment sharing networks using separable temporal exponential random graph models with the Statnet package^78^ in R (The R Foundation). The model is fitted to reproduce observed PWID network characteristics,^28,77^ with dynamic formation and dissolution of partnerships over time based on average partnership duration. Given considerable variation in mean degree across injection networks, we performed analyses in both a sparse network setting (mean degree of 1.4, with lower HCV transmission) and a dense network setting (mean degree of 3, with higher transmission). Initiation, cessation, and relapse of injection drug use were also simulated with transition rates estimated from published literature. Further details are presented in the eAppendix in Supplement 1.

### Natural History of HCV Infection

Individuals can acquire infection from their injection partners. The monthly transmission probability between HCV-discordant partners was previously calibrated to prevalence and incidence data from the SNAP study.^28,77^ We assumed that reinfection risk after cure is approximately one-third of the primary infection risk.^49,50^ Acute HCV infection may spontaneously clear before progressing to chronic HCV infection during the first 6 months of infection.^30^ Fibrosis progression was simulated in individuals with chronic HCV infection.^34,79^

### Mortality

Each PWID was assigned an age-dependent base monthly mortality rate, with excess mortality rates associated with injection drug use, HCV infection, and fibrosis stages (eAppendix in Supplement 1).

### HCV Testing and Treatment

Each PWID was assigned an attribute indicating whether they had opportunity to be tested for HCV infection governed by the parameter of coverage to account for the empirical observation that not all PWID can be reached by HCV testing due to limited access to and/or utilization of HCV care.^35^ Those who can be reached were assigned a monthly testing probability specific to the testing frequency (1 / testing frequency) assessed. For individuals who were diagnosed with a current infection, a cascade of linkage to care (visiting a health care professional), DAA initiation and completion, and sustained virologic response was modeled. We assumed that all PWID were eligible for retreatment if they were reinfected. We simulated 5 HCV testing frequencies: every 2 years, every year, every 6 months, every 3 months, and every month.

### Health Utilities and Costs

We used a health care sector perspective for this analysis. We used age-specific background health utilities based on population norms measured with EQ-5D.^56^ Additional health decrements associated with HCV and with other outcomes relating to injection drug use were captured using a multiplicative model,^80^ with condition-specific utility values reported in the Table (eAppendix in Supplement 1).

We included monthly health care costs related to injection status, HCV infection status, and fibrosis stage for each PWID. Costs associated with testing and treatment cascade were recorded by event. We derived unit costs from the 2021 federal fee schedule when available. All costs were inflated to 2021 US dollars.

### Simulation Setting and Cost-Effectiveness Analysis

Simulations were conducted in monthly time steps. To calculate longer-term outcomes that may be attributed to interventions, we ran interventions for 10 years and then recorded subsequent health and economic outcomes over an additional 50 years, with no HCV testing or treatment and networks closed to new entrants over the extended accounting period. For each testing frequency and sensitivity analysis scenario, we iterated the simulation 500 times (convergence test shown in eFigure 1 in Supplement 1). To reduce Monte Carlo error across comparative runs, we used the same random-number seed for different testing frequencies and scenarios within each iteration.

We computed the cumulative per-person costs and quality-adjusted life-years (QALYs) by dividing the total cumulative costs and QALYs of the simulated PWID population over 60 years by a weighted cohort size estimate, which accounted for individuals entering the cohort at different times: 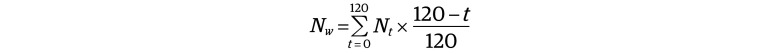,with *t* representing time in months and *N_t_* representing the number of individuals entering the cohort at month *t*. To evaluate cost-effectiveness of different testing frequencies, we calculated the incremental cost-effectiveness ratio (ICER) of each testing frequency by dividing the incremental costs (averaged over iterations) by the QALYs gained (averaged over iterations).^81^ Each testing frequency was compared with the next most effective frequency after eliminating strategies that were dominated (higher costs and lower QALYs) or weakly dominated (higher ICER compared with a more effective strategy). We discounted costs and QALYs at a 3% annual rate.^72^ To characterize uncertainty around the comparative cost-effectiveness of different testing strategies in relation to stochastic variation in the path-dependent evolution of outcomes within a network, we recorded the percentage of iterations in which each testing frequency had the greatest net benefit (total QALYs gained multiplied by willingness to pay [WTP] minus total costs) at different WTP thresholds per QALY.

### Sensitivity and Scenario Analyses

We performed sensitivity analysis on testing coverage, linkage to care rate, transmission probability between injection partners, average partnership duration, and standardized mortality ratios associated with injection drug use, with values for each parameter increased or decreased by 50%. To test the robustness of outcomes to more unfavorable assumptions about test performance and spontaneous clearance, we conducted additional sensitivity analyses using lower-bound values for sensitivity (96.7%^82^) and specificity (99%, expert opinion) of HCV antibody testing and a higher rate of spontaneous clearance (50%).^83^ We also simulated 2 scenarios in which there was ongoing utilization of harm-reduction services, including syringe services programs (SSPs) and medication for opioid use disorder (MOUD), during the 10-year intervention period with either a lower coverage (20% engaged in SSP and 10% engaged in MOUD) or a higher coverage (40% engaged in SSP and 20% engaged in MOUD) to assess how SSP and MOUD coverage affects the cost-effectiveness of HCV testing. People who engage in SSP were assumed to have reduced per-partnership HCV transmission probability and could initiate MOUD; people who engage in MOUD were assumed to cease injection drug use and would not use SSP. In another scenario analysis, we altered HCV reinfection risk after cure to either zero or equal to the primary infection risk.

Results in sensitivity and scenario analyses were summarized in terms of how the changes in parameter values and assumptions altered the ICER for testing every 6 months vs no testing compared with the ICER computed from the main analysis. We show mean and 95% uncertainty intervals for changes in ICER using a bootstrap sample of 500 iterations.

All analyses were conducted using R version 4.3.2. Data were collected from November 2008 to August 2010, and data were analyzed from September 2017 to December 2019.

## Results

The mean initial age of simulated PWID was 32 years. Without testing and treatment for hepatitis C, the cumulative person-years of HCV infections, numbers of new HCV infections, and reinfections in the weighted initial cohort of 1552 PWID over 60 years were 38 576 person-years, 604 new infections, and 112 reinfections, respectively, in the sparse network and 47 703 person-years, 817 new infections, and 177 reinfections, respectively, in the dense network. Cumulative numbers of HCV-related deaths and deaths attributable to injection drug use were 364 and 714, respectively, in the sparse network and 444 and 689, respectively, in the dense network. Discounted QALYs and costs per person cumulated over 60 years were 13.15 and $553 441, respectively, in the sparse network and 12.91 and $557 896, respectively, in the dense network.

**Figure 2.**
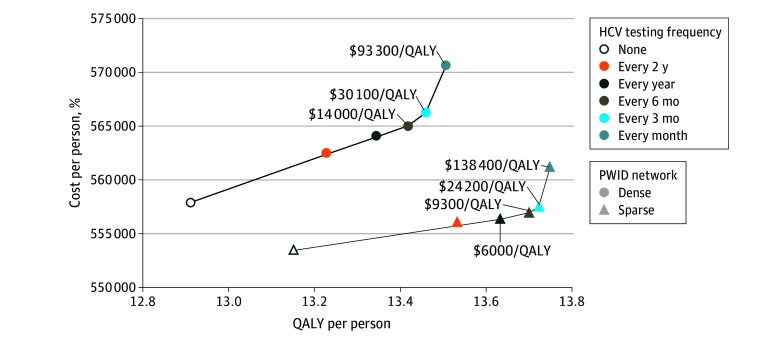
Cumulative Cost, Quality-Adjusted Life-Years (QALYs), and Incremental Cost-Effectiveness Ratios (ICERs) Associated With Different Hepatitis C Virus (HCV) Testing Frequencies in Sparse and Dense People Who Inject Drugs (PWID) Networks Each point shows the cumulative cost and QALY per person over 60 years associated with 10 years’ interventions of different HCV testing frequencies in 2 different PWID networks. The lines connecting the successive points are the cost-effectiveness frontiers.

Compared with no testing, implementing HCV testing for 10 years at frequencies from every 2 years to every month increased QALYs by 2.9% to 4.5% in the sparse network; and by 2.5% to 4.6% in the dense network. For the sparse network, testing every 2 years was weakly dominated; testing every year, every 6 months, every 3 months, and every month had ICERs of $6000 per QALY, $9300 per QALY, $24 200 per QALY, and $138 400 per QALY, respectively. For the dense network, testing every 2 years and every year were both weakly dominated; testing every 6 months, 3 months, and month had ICERs of $14 000 per QALY, $30 100 per QALY, and $93 300 per QALY, respectively (Figure 2; eTable 2 in Supplement 1).

Acknowledging that population health and economic outcomes associated with different strategies may vary across states of the world that evolve over a series of path-dependent stochastic events, we recorded the percentage of iterations in which each testing strategy would be preferred based on a net monetary benefit criterion, given a specified WTP threshold per QALY. At a WTP threshold of $50 000 per QALY, average testing intervals of 6 months and 3 months were substantially more likely to be preferred, eg, in sparse networks, these intervals were optimal in 172 of 500 iterations (34%) and 230 of 500 iterations (46%), respectively, compared with the other strategies. At a WTP threshold of $100 000 per QALY, the probability of preferring testing every month increased to a level similar (148 of 500 iterations [30%]) to that of testing every 6 months (136 of 500 iterations [27%]) and 3 months (182 of 500 iterations [36%]).

**Figure 3.**
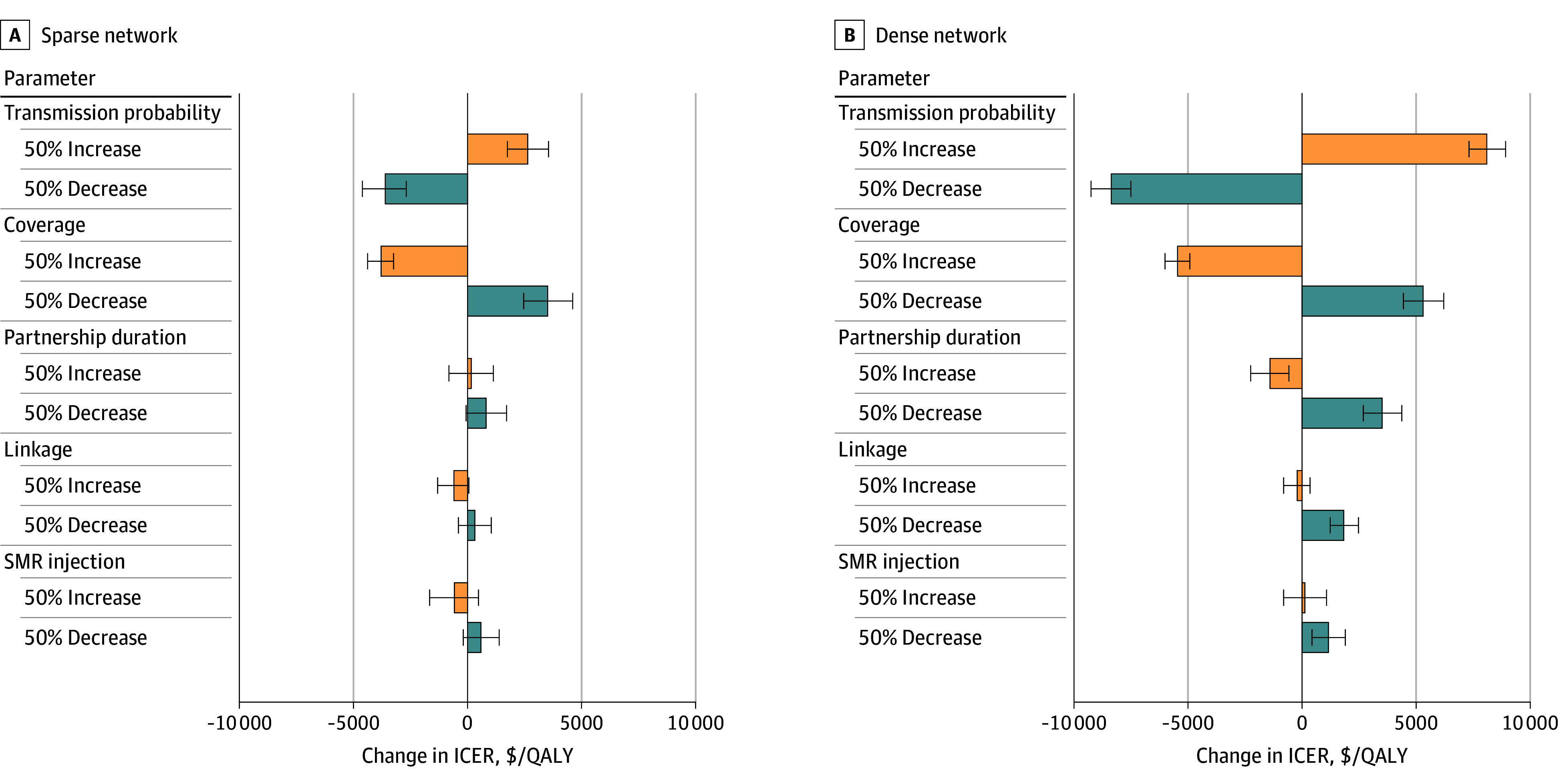
Impact of Key Parameters on Incremental Cost-Effectiveness Ratios (ICERs) of Hepatitis C Virus Testing Every 6 Months vs No Testing in Sparse and Dense Networks Length of each bar shows the average change in ICER with variations in the corresponding parameter from a bootstrap sample of 500 simulation iterations. The error bars show the 95% CIs. QALY indicates quality-adjusted life-year; SMR, standardized mortality ratio.

As shown in Figure 3, HCV transmission probability between injection partners and testing coverage had considerable impact on ICER, with greater influence in the dense network compared with the sparse network. However, no change in parameter values increased ICERs of testing every 6 months vs no testing to more than $20 000 per QALY. Setting antibody test sensitivity and specificity at values that were less favorable than in the base case analyses had little effect on the results, producing ICERs for testing every 3 months that remained less than $25 000 per QALY for both sparse and dense networks. Assuming higher spontaneous clearance rates lowered costs and QALYS overall compared with the base case by less than 0.5%, leading to small changes in ICERs of testing every 3 months (by 3% in sparse networks and 5% in dense networks) but favored lower-frequency testing strategies (eFigure 2 in Supplement 1).

Variation in reinfection risk had a substantial impact on ICERs. When there was no reinfection after cure, the ICERs decreased by $5400 per QALY and $12 700 per QALY in sparse and dense networks, respectively; and when reinfection risk was equal to primary infection risk, the ICERs increased by $10 600 per QALY and $26 400 per QALY in sparse and dense networks, respectively. Combining harm-reduction interventions of SSP and MOUD with lower or higher coverages decreased the ICERs by approximately $2000 per QALY in the sparse network and by $3000 to $5000 per QALY in the dense network.

## Discussion

In this study, we found that HCV testing among PWID every 6 or 12 months had ICERs of less than $15 000 per QALY gained compared with no testing, assuming status quo coverage (60%) and linkage to care (47%) levels and without the addition of SSPs or MOUD. These ICERs would be regarded as indicating very high value for money compared with commonly used benchmarks suggesting that ICERs less than $100 000 to $150 000 per QALY are considered reasonable.^84^ The ICERs were comparable with other cost-effective analyses of different HCV testing programs.^9,85,86^ Although major DAA patents would not expire until 2035 in the US, there might be strategies to reduce their costs earlier,^87^ which could further improve the cost-effectiveness of frequent testing. Testing at higher frequencies—every 3 months in the sparse network or monthly in the dense network—costs more per QALY gained than testing every 6 or 12 months but would still be regarded as cost-effective based on a $100 000 per QALY benchmark. However, the feasibility of high frequencies of testing should be considered, especially for settings where constraints may include not only financial budgets but also health care capacity, as additional resources may be required to implement high-frequency testing (eg, substantial expansion in testing capacity or targeted medical outreach efforts). On the other hand, settings equipped to routinely care for PWID, such as SSPs, may consider more frequent testing. In addition, allocation of limited resources for more frequent testing vs expanded coverage should also be considered.

Cost-effectiveness of testing generally appeared to be more favorable in sparse PWID networks than in dense PWID networks, although testing every month had an ICER that fell below $100 000 per QALY for dense networks but above $100 000 per QALY for sparse networks. Prevalence and incidence of HCV infection are both higher in dense networks, which contribute to lower QALYs and higher costs due to more HCV-related health conditions in the population. This reflects the fact that maintaining the same level of population health outcomes would require more resources to test and treat additional HCV cases in denser networks.

Testing coverage and linkage to care rates are both components of the HCV care cascade, but coverage had substantially higher impact on the cost-effectiveness of HCV testing than linkage to care. A small portion of the difference can be explained by the magnitude of parameter variation in the sensitivity analysis: 50% change of the base case coverage of 60% is greater than that of base case linkage rate of 46%. However, the main reason may be that when coverage is low, there will be a pool of PWID who remain chronically infected and able to transmit HCV no matter how frequently others are tested and treated. Ensuring sufficient testing coverage for PWID is needed to actualize the benefit of more frequent HCV testing. This finding also suggests that point of care testing for PWID could be promising: easy-to-use fingerstick testing can be implemented in a variety of settings, expanding coverage; the quicker results can reduce lost to follow-up in the care cascade. It is possible that more moderate frequency of testing would be preferred because of improved care cascade. Another contextual factor contributing to the care cascade is insurance coverage. Historically, many states imposed strict eligibility criteria for Medicaid coverage of DAAs, eg, severe liver disease. While these restrictions have been gradually lifted over time, they still exist in a number of states.^88^ Strict restrictions on Medicaid coverage, either for primary infection or reinfection, would result in decreased cost-effectiveness.

Cost-effectiveness of testing was most sensitive to values for the HCV transmission probability between injection partners and reinfection risk after cure. Our findings emphasized the importance of maintaining lower transmission risks to improve the cost-effectiveness of frequent HCV testing, which may require extra costs. In analyses that added harm-reduction interventions (SSP and MOUD) with associated effects and costs, the cost-effectiveness of HCV testing also improved, indicating the benefit of combining harm reduction with HCV testing and treatment. Notably, recent increases in polydrug use with stimulants and transition to fentanyl^89^ may reduce adherence to MOUD^90,91^ and diminish the benefits of combining MOUD with HCV testing.

In our model, altering excess mortality ratios associated with injection drug use would change the mortality rate of every individual and have the same effect as changing baseline mortality of the whole simulated population. In reality, baseline mortality of a PWID population can be affected by demographic characteristics and other factors, and our results show that change in mortality in a PWID population would have minimal impact on the cost-effectiveness of HCV testing.

### Limitations

There are several limitations in our study. First, we used data from a clinical trial setting to estimate the monthly health care costs for PWID, which might be high for community-dwelling PWID, who can have lower health care access. This likely overestimated the total health care costs in all scenarios but disproportionally affected the scenarios with high frequencies of testing, since HCV testing and treatment would increase the average lifespan of PWID. If monthly health care costs associated with drug use are lower, frequent HCV testing and treatment can be more attractive. Second, costs of HCV testing can vary between locations and settings, but since these costs are very low compared with other costs, we do not expect them to affect the findings. Third, while we conducted 1-way sensitivity analyses to examine the impact of key model inputs on our findings, we did not assess the joint uncertainty around all input parameter values simultaneously in a probabilistic sensitivity analysis. Given relatively limited variation in outcomes across 1-way sensitivity analyses and a paucity of empirical data to support definition of the joint distribution of parameter values, we did not expect that a probabilistic sensitivity analysis would contribute additional insights.

## Conclusions

The findings in this economic evaluation study indicate that HCV testing among PWID every 6 to 12 months or even more frequently could be a reasonable, cost-effective strategy for a community to implement or an advisory committee to recommend. Increasing access to and utilization of HCV care among PWID and incorporating harm-reduction services, such as SSP and MOUD, would further improve the cost-effectiveness.

## Notes

Supplement 1. **eTable 1.** Literature Review of Urban PWID Networks in the US **eTable 2.** Cumulative Cost, QALY, and Incremental Cost-Effectiveness Ratio (ICER) of Different HCV Testing Frequencies Among People Who Inject Drugs (PWID) Networks **eFigure 1.** Average Cost, QALY, and ICER (Compared With No Testing) of Each Testing Frequency Over Different Number of Iterations **eFigure 2.** Impact of HCV Antibody Testing Characteristics and Spontaneous Clearance Rate on the Cumulative Cost, QALY, and Incremental Cost-Effectiveness Ratio (ICER) of Different HCV Testing Frequencies Among People Who Inject Drugs (PWID) Networks
eAppendix.

eReferences.

Supplement 2. Data Sharing Statement

## References

[1] Gane EJ, Stedman CA, Hyland RH, . Nucleotide polymerase inhibitor sofosbuvir plus ribavirin for hepatitis C. N Engl J Med. 2013;368(1):34-44. doi:10.1056/NEJMoa1208953 23281974

[2] World Health Organization. Combating Hepatitis B and C to Reach Elimination by 2030: Advocacy Brief. World Health Organization; 2016.

[3] US Department of Health and Human Services. Viral Hepatitis: National Strategic Plan—A Roadmap to Elimination for the United States, 2021-2025. Department of Health and Human Services; 2021.

[4] Lewis KC, Barker LK, Jiles RB, Gupta N. Estimated prevalence and awareness of hepatitis C virus infection among US adults: National Health and Nutrition Examination Survey, January 2017-March 2020. Clin Infect Dis. 2023;77(10):1413-1415. doi:10.1093/cid/ciad411 37417196 PMC11000503

[5] Schillie S, Wester C, Osborne M, Wesolowski L, Ryerson AB. CDC recommendations for hepatitis C screening among adults - United States, 2020. MMWR Recomm Rep. 2020;69(2):1-17. doi:10.15585/mmwr.rr6902a1 32271723 PMC7147910

[6] Owens DK, Davidson KW, Krist AH, ; US Preventive Services Task Force. Screening for hepatitis C virus infection in adolescents and adults: US Preventive Services Task Force recommendation statement. JAMA. 2020;323(10):970-975. doi:10.1001/jama.2020.1123 32119076

[7] Scott N, Iser DM, Thompson AJ, Doyle JS, Hellard ME. Cost-effectiveness of treating chronic hepatitis C virus with direct-acting antivirals in people who inject drugs in Australia. J Gastroenterol Hepatol. 2016;31(4):872-882. doi:10.1111/jgh.13223 26514998

[8] van Santen DK, de Vos AS, Matser A, . Cost-effectiveness of hepatitis C treatment for people who inject drugs and the impact of the type of epidemic; extrapolating from Amsterdam, the Netherlands. PLoS One. 2016;11(10):e0163488. doi:10.1371/journal.pone.0163488 27711200 PMC5053429

[9] Barbosa C, Fraser H, Hoerger TJ, . Cost-effectiveness of scaling-up HCV prevention and treatment in the United States for people who inject drugs. Addiction. 2019;114(12):2267-2278. doi:10.1111/add.14731 31307116 PMC7751348

[10] Martin NK, Hickman M, Miners A, Hutchinson SJ, Taylor A, Vickerman P. Cost-effectiveness of HCV case-finding for people who inject drugs via dried blood spot testing in specialist addiction services and prisons. BMJ Open. 2013;3(8):e003153. doi:10.1136/bmjopen-2013-003153 23943776 PMC3752052

[11] Helsper CW, Janssen MP, van Essen GA, . Effectiveness and cost-effectiveness of nationwide campaigns for awareness and case finding of hepatitis C targeted at people who inject drugs and the general population in the Netherlands. Int J Drug Policy. 2017;47:117-125. doi:10.1016/j.drugpo.2017.07.022 28826994

[12] Girardin F, Hearmon N, Negro F, Eddowes L, Bruggmann P, Castro E. Increasing hepatitis C virus screening in people who inject drugs in Switzerland using rapid antibody saliva and dried blood spot testing: a cost-effectiveness analysis. J Viral Hepat. 2019;26(2):236-245. doi:10.1111/jvh.13023 30338887

[13] Duchesne L, Hejblum G, Toure Kane NC, . Model-based cost-effectiveness estimates of testing strategies for diagnosing hepatitis C virus infection in people who use injecting drugs in Senegal. Int J Drug Policy. 2020;75:102613. doi:10.1016/j.drugpo.2019.102613 31786434

[14] Cousien A, Tran VC, Deuffic-Burban S, . Effectiveness and cost-effectiveness of interventions targeting harm reduction and chronic hepatitis C cascade of care in people who inject drugs: the case of France. J Viral Hepat. 2018;25(10):1197-1207. doi:10.1111/jvh.12919 29660211

[15] Mabileau G, Scutelniciuc O, Tsereteli M, . Intervention packages to reduce the impact of HIV and HCV infections among people who inject drugs in Eastern Europe and Central Asia: a modeling and cost-effectiveness study. Open Forum Infect Dis. 2018;5(3):ofy040. doi:10.1093/ofid/ofy040 29594179 PMC5861407

[16] Gutkind S, Schackman BR, Morgan JR, . Cost-effectiveness of hepatitis C virus treatment models for people who inject drugs in opioid agonist treatment programs. Clin Infect Dis. 2020;70(7):1397-1405. doi:10.1093/cid/ciz384 31095683 PMC7318779

[17] Stevens ER, Nucifora KA, Hagan H, . Cost-effectiveness of direct antiviral agents for hepatitis C virus infection and a combined intervention of syringe access and medication-assisted therapy for opioid use disorders in an injection drug use population. Clin Infect Dis. 2020;70(12):2652-2662. doi:10.1093/cid/ciz726 31400755 PMC7286369

[18] Ward Z, Reynolds R, Campbell L, . Cost-effectiveness of the HepCATT intervention in specialist drug clinics to improve case-finding and engagement with HCV treatment for people who inject drugs in England. Addiction. 2020;115(8):1509-1521. doi:10.1111/add.14978 31984606 PMC10762643

[19] Mafirakureva N, Stone J, Fraser H, . An intensive model of care for hepatitis C virus screening and treatment with direct-acting antivirals in people who inject drugs in Nairobi, Kenya: a model-based cost-effectiveness analysis. Addiction. 2022;117(2):411-424. doi:10.1111/add.15630 34184794 PMC8737065

[20] Marquez LK, Fleiz C, Burgos JL, . Cost-effectiveness of hepatitis C virus (HCV) elimination strategies among people who inject drugs (PWID) in Tijuana, Mexico. Addiction. 2021;116(10):2734-2745. doi:10.1111/add.15456 33620750 PMC8380744

[21] Hickman M, De Angelis D, Vickerman P, Hutchinson S, Martin NK. Hepatitis C virus treatment as prevention in people who inject drugs: testing the evidence. Curr Opin Infect Dis. 2015;28(6):576-582. doi:10.1097/QCO.0000000000000216 26524330 PMC4659818

[22] Metzig C, Surey J, Francis M, Conneely J, Abubakar I, White PJ. Impact of hepatitis C treatment as prevention for people who inject drugs is sensitive to contact network structure. Sci Rep. 2017;7(1):1833. doi:10.1038/s41598-017-01862-6 28500290 PMC5431870

[23] Fuller CM, Ompad DC, Galea S, Wu Y, Koblin B, Vlahov D. Hepatitis C incidence–a comparison between injection and noninjection drug users in New York City. J Urban Health. 2004;81(1):20-24. doi:10.1093/jurban/jth084 15047780 PMC3456148

[24] Shah NG, Galai N, Celentano DD, Vlahov D, Strathdee SA. Longitudinal predictors of injection cessation and subsequent relapse among a cohort of injection drug users in Baltimore, MD, 1988-2000. Drug Alcohol Depend. 2006;83(2):147-156. doi:10.1016/j.drugalcdep.2005.11.007 16364568

[25] Evans E, Li L, Min J, . Mortality among individuals accessing pharmacological treatment for opioid dependence in California, 2006-10. Addiction. 2015;110(6):996-1005. doi:10.1111/add.12863 25644938 PMC4452110

[26] Bruno S, Zuin M, Crosignani A, . Predicting mortality risk in patients with compensated HCV-induced cirrhosis: a long-term prospective study. Am J Gastroenterol. 2009;104(5):1147-1158. doi:10.1038/ajg.2009.31 19352340

[27] van der Meer AJ, Veldt BJ, Feld JJ, . Association between sustained virological response and all-cause mortality among patients with chronic hepatitis C and advanced hepatic fibrosis. JAMA. 2012;308(24):2584-2593. doi:10.1001/jama.2012.144878 23268517

[28] Zhu L, Thompson WW, Hagan L, . Potential impact of curative and preventive interventions toward hepatitis C elimination in people who inject drugs—a network modeling study. Int J Drug Policy. 2024;130:104539. doi:10.1016/j.drugpo.2024.104539 39033645 PMC11347083

[29] Morris MD, Evans J, Montgomery M, . Intimate injection partnerships are at elevated risk of high-risk injecting: a multi-level longitudinal study of HCV-serodiscordant injection partnerships in San Francisco, CA. PLoS One. 2014;9(10):e109282. doi:10.1371/journal.pone.0109282 25286346 PMC4186818

[30] Smith DJ, Jordan AE, Frank M, Hagan H. Spontaneous viral clearance of hepatitis C virus (HCV) infection among people who inject drugs (PWID) and HIV-positive men who have sex with men (HIV+ MSM): a systematic review and meta-analysis. BMC Infect Dis. 2016;16(1):471. doi:10.1186/s12879-016-1807-5 27595855 PMC5011802

[31] Busch MP, Page Shafer KA. Acute-Phase Hepatitis C Virus Infection: Implications for Research, Diagnosis, and Treatment. The University of Chicago Press; 2005:959-961. 10.1086/428583 15824986

[32] Kim HN, Corcorran MA. Diagnosis of acute HCV infection. Accessed March 17, 2023. https://www.hepatitisc.uw.edu/go/screening-diagnosis/acute-diagnosis/core-concept/all

[33] Stramer SL, Glynn SA, Kleinman SH, ; National Heart, Lung, and Blood Institute Nucleic Acid Test Study Group. Detection of HIV-1 and HCV infections among antibody-negative blood donors by nucleic acid-amplification testing. N Engl J Med. 2004;351(8):760-768. doi:10.1056/NEJMoa040085 15317889

[34] Erman A, Krahn MD, Hansen T, . Estimation of fibrosis progression rates for chronic hepatitis C: a systematic review and meta-analysis update. BMJ Open. 2019;9(11):e027491. doi:10.1136/bmjopen-2018-027491 31719068 PMC6858137

[35] Smith A, Xia SC, Sionean C, . HIV Infection, risk, prevention, and testing behaviors among persons who inject drugs: National HIV Behavioral Surveillance: injection drug use, 23 US cities, 2018. Accessed January 17, 2022. https://stacks.cdc.gov/view/cdc/106349

[36] Barocas JA, Brennan MB, Hull SJ, Stokes S, Fangman JJ, Westergaard RP. Barriers and facilitators of hepatitis C screening among people who inject drugs: a multi-city, mixed-methods study. Harm Reduct J. 2014;11(1):1-8. doi:10.1186/1477-7517-11-1 24422784 PMC3896714

[37] Hajarizadeh B, Kairouz A, Ottaviano S, . Global, regional, and country-level coverage of testing and treatment for HIV and hepatitis C infection among people who inject drugs: a systematic review. Lancet Glob Health. 2023;11(12):e1885-e1898. doi:10.1016/S2214-109X(23)00461-8 37973339

[38] US Food and Drug Administration. Complete list of donor screening assays for infectious agents and HIV diagnostic assays. Accessed April 24, 2024. https://www.fda.gov/vaccines-blood-biologics/complete-list-donor-screening-assays-infectious-agents-and-hiv-diagnostic-assays

[39] Viner K, Kuncio D, Newbern EC, Johnson CC. The continuum of hepatitis C testing and care. Hepatology. 2015;61(3):783-789. doi:10.1002/hep.27584 25348499

[40] Falade-Nwulia O, Ward KM, McCormick S, . Network-based recruitment of people who inject drugs for hepatitis C testing and linkage to care. J Viral Hepat. 2020;27(7):663-670. doi:10.1111/jvh.13274 32045086 PMC7299737

[41] Seña AC, Willis SJ, Hilton A, . Efforts at the frontlines: implementing a hepatitis C testing and linkage-to-care program at the local public health level. Public Health Rep. 2016;131(suppl 2):57-64. doi:10.1177/00333549161310S210 27168663 PMC4853330

[42] Harris AM, Khan MA, Osinubi A, Nelson NP, Thompson WW. Hepatitis C treatment among commercially or Medicaid-insured individuals, 2014-2018. Am J Prev Med. 2021;61(5):716-723. doi:10.1016/j.amepre.2021.05.017 34362617

[43] Younossi ZM, Bacon BR, Dieterich DT, . Disparate access to treatment regimens in chronic hepatitis C patients: data from the TRIO network. J Viral Hepat. 2016;23(6):447-454. doi:10.1111/jvh.12506 26840452

[44] Berg T, Naumann U, Stoehr A, . Real-world effectiveness and safety of glecaprevir/pibrentasvir for the treatment of chronic hepatitis C infection: data from the German Hepatitis C-Registry. Aliment Pharmacol Ther. 2019;49(8):1052-1059. doi:10.1111/apt.15222 30874328

[45] Cornberg M, Stoehr A, Naumann U, . Real-world safety, effectiveness, and patient-reported outcomes in patients with chronic hepatitis C virus infection treated with glecaprevir/pibrentasvir: updated data from the German Hepatitis C-Registry (DHC-R). Viruses. 2022;14(7):1541. doi:10.3390/v14071541 35891520 PMC9318383

[46] Sowah LA, Smeaton L, Brates I, . Perspectives on adherence from the ACTG 5360 MINMON trial: a minimum monitoring approach with 12 weeks of sofosbuvir/velpatasvir in chronic hepatitis C treatment. Clin Infect Dis. 2023;76(11):1959-1968. doi:10.1093/cid/ciad034 36694361 PMC10249990

[47] Hajarizadeh B, Cunningham EB, Reid H, Law M, Dore GJ, Grebely J. Direct-acting antiviral treatment for hepatitis C among people who use or inject drugs: a systematic review and meta-analysis. Lancet Gastroenterol Hepatol. 2018;3(11):754-767. doi:10.1016/S2468-1253(18)30304-2 30245064

[48] Cunningham EB, Amin J, Feld JJ, ; SIMPLIFY study group. Adherence to sofosbuvir and velpatasvir among people with chronic HCV infection and recent injection drug use: the SIMPLIFY study. Int J Drug Policy. 2018;62:14-23. doi:10.1016/j.drugpo.2018.08.013 30352330

[49] Hajarizadeh B, Cunningham EB, Valerio H, . Hepatitis C reinfection after successful antiviral treatment among people who inject drugs: a meta-analysis. J Hepatol. 2020;72(4):643-657. doi:10.1016/j.jhep.2019.11.012 31785345

[50] Esmaeili A, Mirzazadeh A, Carter GM, . Higher incidence of HCV in females compared to males who inject drugs: a systematic review and meta-analysis. J Viral Hepat. 2017;24(2):117-127. doi:10.1111/jvh.12628 27790803 PMC5239758

[51] Valencia J, Alvaro-Meca A, Troya J, . High rates of early HCV reinfection after DAA treatment in people with recent drug use attended at mobile harm reduction units. Int J Drug Policy. 2019;72:181-188. doi:10.1016/j.drugpo.2019.06.016 31253391

[52] Caven M, Malaguti A, Robinson E, Fletcher E, Dillon JF. Impact of Hepatitis C treatment on behavioural change in relation to drug use in people who inject drugs: a systematic review. Int J Drug Policy. 2019;72:169-176. doi:10.1016/j.drugpo.2019.05.011 31109776

[53] Platt L, Minozzi S, Reed J, . Needle and syringe programmes and opioid substitution therapy for preventing HCV transmission among people who inject drugs: findings from a Cochrane Review and meta-analysis. Addiction. 2018;113(3):545-563. doi:10.1111/add.14012 28891267 PMC5836947

[54] Gindi RM, Rucker MG, Serio-Chapman CE, Sherman SG. Utilization patterns and correlates of retention among clients of the needle exchange program in Baltimore, Maryland. Drug Alcohol Depend. 2009;103(3):93-98. doi:10.1016/j.drugalcdep.2008.12.018 19464827 PMC2744092

[55] Krawczyk N, Williams AR, Saloner B, Cerdá M. Who stays in medication treatment for opioid use disorder? a national study of outpatient specialty treatment settings. J Subst Abuse Treat. 2021;126:108329. doi:10.1016/j.jsat.2021.108329 34116820 PMC8197774

[56] Hanmer J, Lawrence WF, Anderson JP, Kaplan RM, Fryback DG. Report of nationally representative values for the noninstitutionalized US adult population for 7 health-related quality-of-life scores. Med Decis Making. 2006;26(4):391-400. doi:10.1177/0272989X06290497 16855127

[57] Pyne JM, French M, McCollister K, Tripathi S, Rapp R, Booth B. Preference-weighted health-related quality of life measures and substance use disorder severity. Addiction. 2008;103(8):1320-1329. doi:10.1111/j.1360-0443.2008.02153.x 18397359 PMC3767314

[58] Saeed YA, Phoon A, Bielecki JM, . A systematic review and meta-analysis of health utilities in patients with chronic hepatitis C. Value Health. 2020;23(1):127-137. doi:10.1016/j.jval.2019.07.005 31952667

[59] McCollister KE, Leff JA, Yang X, . Cost of pharmacotherapy for opioid use disorders following inpatient detoxification. Am J Manag Care. 2018;24(11):526-531. 30452209 PMC6345513

[60] Murphy SM, McCollister KE, Leff JA, . Cost-effectiveness of buprenorphine–naloxone versus extended-release naltrexone to prevent opioid relapse. Ann Intern Med. 2019;170(2):90-98. doi:10.7326/M18-0227 30557443 PMC6581635

[61] Lee JD, Nunes EV, Mpa PN, . NIDA Clinical Trials Network CTN-0051, Extended-Release Naltrexone vs. Buprenorphine for Opioid Treatment (X:BOT): study design and rationale. Contemp Clin Trials. 2016;50:253-264. doi:10.1016/j.cct.2016.08.004 27521809 PMC5416469

[62] Chhatwal J, Ferrante SA, Brass C, . Cost-effectiveness of boceprevir in patients previously treated for chronic hepatitis C genotype 1 infection in the United States. Value Health. 2013;16(6):973-986. doi:10.1016/j.jval.2013.07.006 24041347 PMC3820000

[63] McAdam-Marx C, McGarry LJ, Hane CA, Biskupiak J, Deniz B, Brixner DI. All-cause and incremental per patient per year cost associated with chronic hepatitis C virus and associated liver complications in the United States: a managed care perspective. J Manag Care Pharm. 2011;17(7):531-546. doi:10.18553/jmcp.2011.17.7.531 21870894 PMC10438304

[64] Davis KL, Mitra D, Medjedovic J, Beam C, Rustgi V. Direct economic burden of chronic hepatitis C virus in a United States managed care population. J Clin Gastroenterol. 2011;45(2):e17-e24. doi:10.1097/MCG.0b013e3181e12c09 20628308

[65] US Centers for Medicare & Medicaid Services. Clinical diagnostic laboratory fee schedule. Accessed March 17, 2023. https://www.cms.gov/medicare/payment/fee-schedules/clinical-laboratory-fee-schedule-clfs

[66] Schackman BR, Gutkind S, Morgan JR, . Cost-effectiveness of hepatitis C screening and treatment linkage intervention in US methadone maintenance treatment programs. Drug Alcohol Depend. 2018;185:411-420. doi:10.1016/j.drugalcdep.2017.11.031 29477574 PMC5889754

[67] American Association for the Study of Liver Diseases. Simplified HCV treatment for treatment-naive adults without cirrhosis. Accessed October 24, 2022. https://www.hcvguidelines.org/treatment-naive/simplified-treatment.

[68] US Centers for Medicare & Medicaid Services. 2021 Physician Fee Schedule. Accessed March 17, 2023. https://www.cms.gov/medicare/payment/fee-schedules/physician

[69] Office of Procurement Acquisition and Logistics. Pharmaceutical prices. Accessed March 28, 2023. https://www.va.gov/opal/nac/fss/pharmprices.asp

[70] Teshale EH, Asher A, Aslam MV, . Estimated cost of comprehensive syringe service program in the United States. PLoS One. 2019;14(4):e0216205. doi:10.1371/journal.pone.0216205 31026295 PMC6485753

[71] Fairley M, Humphreys K, Joyce VR, . Cost-effectiveness of treatments for opioid use disorder. JAMA Psychiatry. 2021;78(7):767-777. doi:10.1001/jamapsychiatry.2021.0247 33787832 PMC8014209

[72] Sanders GD, Neumann PJ, Basu A, . Recommendations for conduct, methodological practices, and reporting of cost-effectiveness analyses: second panel on cost-effectiveness in health and medicine. JAMA. 2016;316(10):1093-1103. doi:10.1001/jama.2016.12195 27623463

[73] Havens JR, Lofwall MR, Frost SD, Oser CB, Leukefeld CG, Crosby RA. Individual and network factors associated with prevalent hepatitis C infection among rural Appalachian injection drug users. Am J Public Health. 2013;103(1):e44-e52. doi:10.2105/AJPH.2012.300874 23153148 PMC3518360

[74] Young AM, Jonas AB, Mullins UL, Halgin DS, Havens JR. Network structure and the risk for HIV transmission among rural drug users. AIDS Behav. 2013;17(7):2341-2351. doi:10.1007/s10461-012-0371-2 23184464 PMC3600060

[75] Young AM, Rudolph AE, Quillen D, Havens JR. Spatial, temporal and relational patterns in respondent-driven sampling: evidence from a social network study of rural drug users. J Epidemiol Community Health. 2014;68(8):792-798. doi:10.1136/jech-2014-203935 24692631 PMC4410836

[76] Young AM, Rudolph AE, Havens JR. Network-based research on rural opioid use: an overview of methods and lessons learned. Curr HIV/AIDS Rep. 2018;15(2):113-119. doi:10.1007/s11904-018-0391-2 29457200 PMC5884725

[77] Zhu L, Havens JR, Rudolph AE, . Hepatitis C virus transmission among people who inject drugs in rural United States: mathematical modeling study using stochastic agent-based network simulation (AJE-00824-2024). Am J Epidemiol. Published online March 10, 2025. doi:10.1093/aje/kwaf052 40069947 PMC13066326

[78] Krivitsky PN, Handcock MS, Hunter DR, et al; Statnet. Statnet: Software Tools for the Statistical Modeling of Network Data. Accessed June 25, 2024. http://statnet.org

[79] Smith DJ, Combellick J, Jordan AE, Hagan H. Hepatitis C virus (HCV) disease progression in people who inject drugs (PWID): a systematic review and meta-analysis. Int J Drug Policy. 2015;26(10):911-921. doi:10.1016/j.drugpo.2015.07.004 26298331 PMC4577462

[80] Ara R, Brazier J. Estimating health state utility values for comorbidities. Pharmacoeconomics. 2017;35(suppl 1):89-94. doi:10.1007/s40273-017-0551-z 29052158

[81] Stinnett AA, Paltiel AD. Estimating CE ratios under second-order uncertainty: the mean ratio versus the ratio of means. Med Decis Making. 1997;17(4):483-489. doi:10.1177/0272989X9701700414 9343807

[82] Colin C, Lanoir D, Touzet S, Meyaud-Kraemer L, Bailly F, Trepo C; HEPATITIS Group. Sensitivity and specificity of third-generation hepatitis C virus antibody detection assays: an analysis of the literature. J Viral Hepat. 2001;8(2):87-95. doi:10.1046/j.1365-2893.2001.00280.x 11264728

[83] Seo S, Silverberg MJ, Hurley LB, . Prevalence of spontaneous clearance of hepatitis C virus infection doubled from 1998 to 2017. Clin Gastroenterol Hepatol. 2020;18(2):511-513. doi:10.1016/j.cgh.2019.04.035 31009792 PMC6801008

[84] Neumann PJ, Cohen JT. Measuring the value of prescription drugs. N Engl J Med. 2015;373(27):2595-2597. doi:10.1056/NEJMp1512009 26580666

[85] Schackman BR, Leff JA, Barter DM, . Cost-effectiveness of rapid hepatitis C virus (HCV) testing and simultaneous rapid HCV and HIV testing in substance abuse treatment programs. Addiction. 2015;110(1):129-143. doi:10.1111/add.12754 25291977 PMC4270906

[86] Ward Z, Campbell L, Surey J, . The cost-effectiveness of an HCV outreach intervention for at-risk populations in London, UK. J Antimicrob Chemother. 2019;74(suppl 5):v5-v16. doi:10.1093/jac/dkz451 31782503 PMC6883400

[87] Tu SS, Kottilil S, Mattingly TJ II. Leveraging old hepatitis C therapies. N Engl J Med. 2024;392(1):1-4. doi:10.1056/NEJMp2409842 39752558

[88] Furukawa NW, Ingber SZ, Symum H, . Medicaid expansion and restriction policies for hepatitis C treatment. JAMA Netw Open. 2024;7(7):e2422406. doi:10.1001/jamanetworkopen.2024.22406 39012632 PMC11252896

[89] Ciccarone D. The rise of illicit fentanyls, stimulants and the fourth wave of the opioid overdose crisis. Curr Opin Psychiatry. 2021;34(4):344-350. doi:10.1097/YCO.0000000000000717 33965972 PMC8154745

[90] Foot C, Korthuis PT, Tsui JI, Luo SX, Chan B, Cook RR. Associations between stimulant use and return to illicit opioid use following initiation onto medication for opioid use disorder. Addiction. 2024;119(1):149-157. doi:10.1111/add.16334 37712113 PMC11139042

[91] Socias ME, Wood E, Le Foll B, ; OPTIMA Research Group within the Canadian Research Initiative in Substance Misuse. Impact of fentanyl use on initiation and discontinuation of methadone and buprenorphine/naloxone among people with prescription-type opioid use disorder: secondary analysis of a Canadian treatment trial. Addiction. 2022;117(10):2662-2672. doi:10.1111/add.15954 35712892 PMC9969999

