# Supplementary material for: Health and Economic Impact of Periodic Hepatitis C Virus Testing Among People Who Inject Drugs
Source: JAMA Health Forum. 2025 Jul 3;6(7):e251870. doi: 10.1001/jamahealthforum.2025.1870 (PMC12232219; doi:10.1001/jamahealthforum.2025.1870)
Supplement: Supplement 1. — eTable 1. Literature Review of Urban PWID Networks in the US eTable 2. Cumulative Cost, QALY, and Incremental Cost-Effectiveness Ratio (ICER) of Different HCV Testing Frequencies Among People Who Inject Drugs (PWID) Networks eFigure 1. Average Cost, QALY, and ICER (Compared With No Testing) of Each Testing Frequency Over Different Number of Iterations eFigure 2. Impact of HCV Antibody Testing Characteristics and Spontaneous Clearance Rate on the Cumulative Cost, QALY, and Incremental Cost-Effectiveness Ratio (ICER) of Different HCV Testing Frequencies Among People Who Inject Drugs (PWID) Networks eAppendix. eReferences. [file jamahealthforum-e251870-s001.pdf]

Supplemental Online Content

Zhu L, Furukawa NW, Thompson WW, et al. Health and economic impact of periodic hepatitis C virus testing among people who inject drugs. *JAMA Health Forum.* 2025;6(7):e251870. doi:10.1001/jamahealthforum.2025.1870

eTable 1. Literature Review of Urban PWID Networks in the US
eTable 2. Cumulative Cost, QALY, and Incremental Cost-Effectiveness Ratio (ICER) of Different HCV Testing Frequencies Among People Who Inject Drugs (PWID) Networks
eFigure 1. Average Cost, QALY, and ICER (Compared With No Testing) of Each Testing Frequency Over Different Number of Iterations
eFigure 2. Impact of HCV Antibody Testing Characteristics and Spontaneous Clearance Rate on the Cumulative Cost, QALY, and Incremental Cost-Effectiveness Ratio (ICER) of Different HCV Testing Frequencies Among People Who Inject Drugs (PWID) Networks
eAppendix.
eReferences.

This supplementary material has been provided by the authors to give readers additional information about their work.

© 2025 Zhu L et al. *JAMA Health Forum*.

eTable 1. Literature Review of Urban PWID Networks in the US

Location	Study time	Sample size	Definition of partner	Time counted	Mean degree	HCV sero prevalence	Reference
Chicago (young)	2012-2013	164	Inject most often with	past 6 months	3.0	5.1%	[1]
San Juan	2012	512	Use needle/works after they injected with it	past 12 months	2.7/6.3	48%	[2]
Chicago, Washington DC	1995	123	Injection and sexual partner	previous 30 days	17.4	NA	[3]
San Francisco	2005	477	Know other IDU	last 6 months	18.0-26.0	59.5%	[4]
Baltimore (drug user)	2001-2003	742	Do drugs with	NA	2.2	NA	[5]
Baltimore	1991-1992	293	Do drugs with	last 6 months	5.2	NA	[6]
Baltimore	1993-1994	499	Share drugs	previous 6 months	3.7	NA	[7]
Hartford, CT	2012-2013	528	Inject at the same time and location	previous 6 months	4.2	57.6%	[8]

* Considering our definition of partnerships is sharing needles/syringes or other equipment to prepare drugs in the past six months, we selected a mean degree of 3 from these results for our simulation of dense PWID networks.

© 2025 Zhu L et al. *JAMA Health Forum*.

eTable 2. Cumulative Cost, QALY, and Incremental Cost-Effectiveness Ratio (ICER) of Different HCV Testing Frequencies Among People Who Inject Drugs (PWID) Networks

network	frequency	cost test treat ($)	cost injection ($)	cost liver ($)	cost ($)	QALY	ICER	undiscounted cost ($)	undiscounted QALY
dense	none	-	470,855	87,041	557,896	12.91	-	969,208	22.43
	every 2 years	7,009	477,111	78,372	562,492	13.23	weakly dominated	979,929	23.25
	every year	9,382	479,389	75,294	564,065	13.34	weakly dominated	983,943	23.55
	every 6 months	11,272	480,735	73,009	565,016	13.42	14,037	986,131	23.73
	every 3 months	13,013	481,579	71,693	566,285	13.46	30,109	988,282	23.83
	every month	17,422	482,587	70,634	570,643	13.51	93,321	994,183	23.94
sparse	none	-	474,781	78,660	553,441	13.15	-	965,801	23.12
	every 2 years	5,491	482,300	68,251	556,041	13.53	weakly dominated	973,872	24.12
	every year	6,975	484,152	65,202	556,329	13.63	5,982	975,366	24.38
	every 6 months	8,152	485,494	63,309	556,955	13.70	9,286	977,074	24.55
	every 3 months	9,463	485,707	62,294	557,464	13.72	24,195	977,383	24.60
	every month	13,464	486,229	61,466	561,159	13.75	138,430	982,294	24.67

© 2025 Zhu L et al. *JAMA Health Forum*.

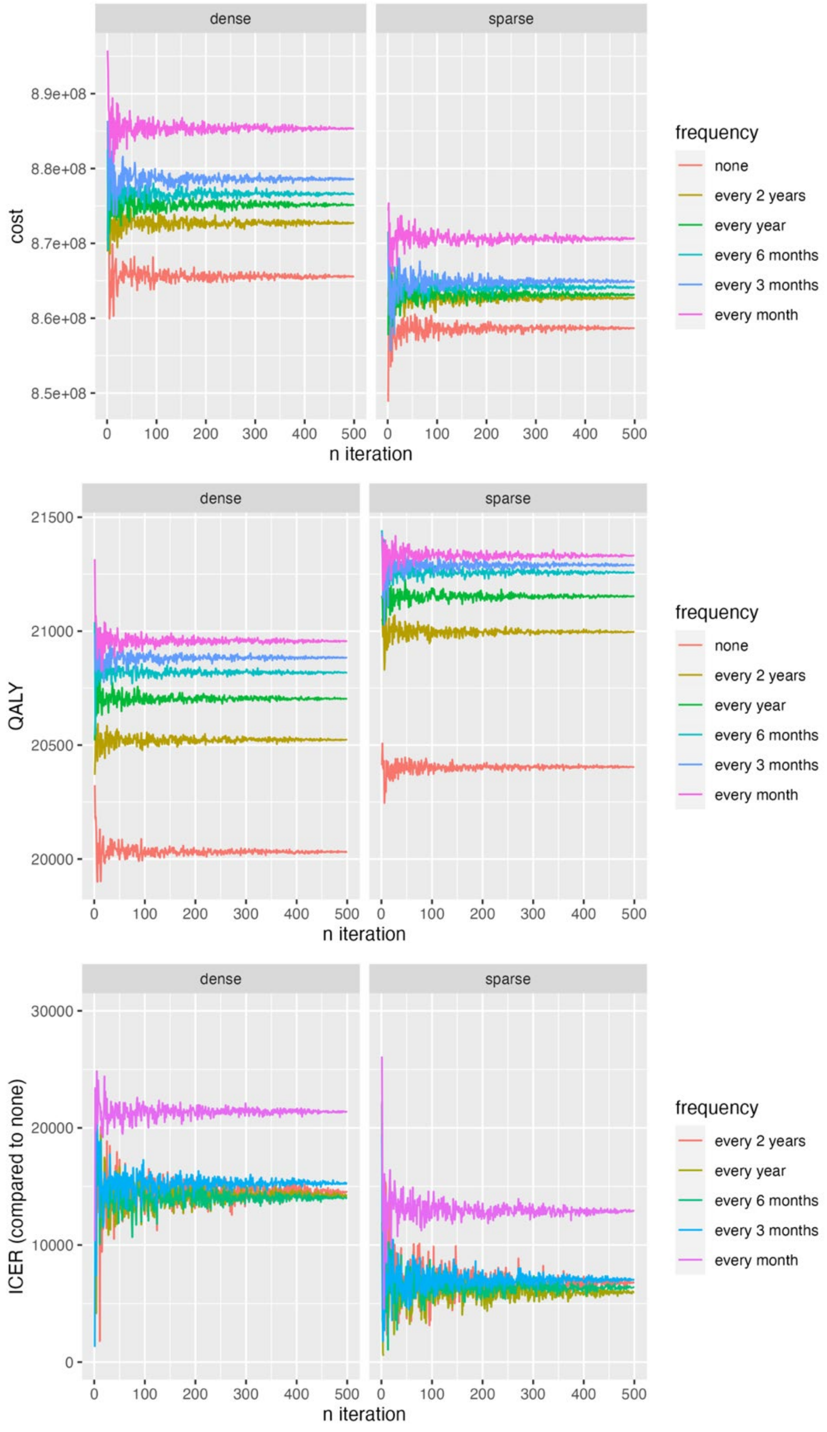

eFigure 1. Average Cost, QALY, and ICER (Compared With No Testing) of Each Testing Frequency Over Different Number of Iterations

© 2025 Zhu L et al. *JAMA Health Forum*.

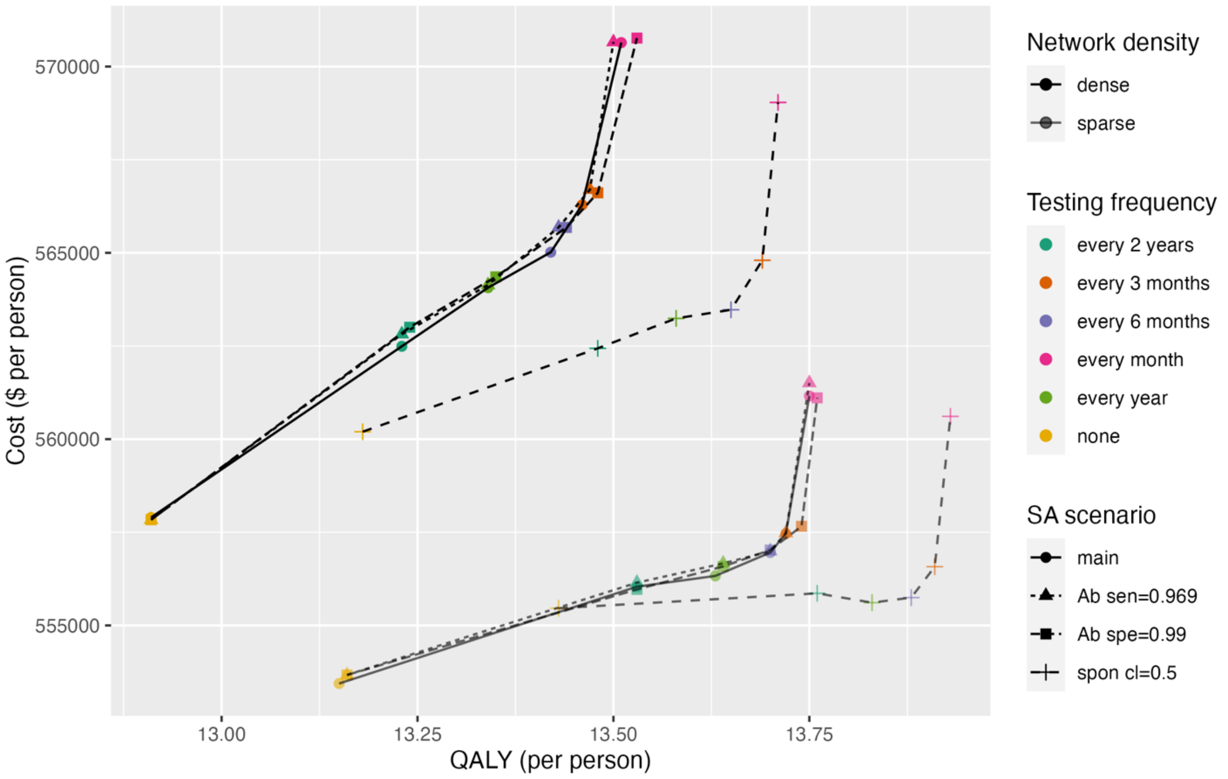

eFigure 2. Impact of HCV Antibody Testing Characteristics and Spontaneous Clearance Rate on the Cumulative Cost, QALY, and Incremental Cost-Effectiveness Ratio (ICER) of Different HCV Testing Frequencies Among People Who Inject Drugs (PWID) Networks

Each point shows the cumulative cost (y-axis) and QALY (x-axis) per person over 60 years associated with 10 years' interventions of different HCV testing frequencies (color label) in two different PWID networks (point shapes) with the main analysis scenario and three sensitivity analysis scenarios (Antibody sensitivity = 0.969, Antibody specificity = 0.99, and spontaneous clearance rate = 50%). The lines connecting the successive points are the cost-effectiveness frontiers.

© 2025 Zhu L et al. *JAMA Health Forum*.

eAppendix.

1. Age distributions

1.1 Initial network and migrated individuals

We fitted normal, lognormal, gamma and Weibull distributions to the age pattern observed in the SNAP PWID sample and selected the lognormal distribution as the best fitting result based on AIC and BIC values.

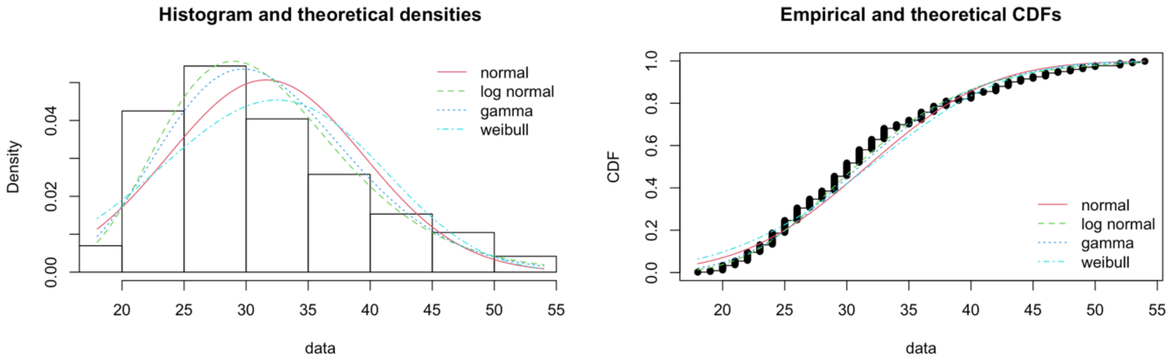

We used the fitted distribution (mean log=3.424, sd log=0.241) truncating at the minimum age of injection initiation = 13 to assign age distributions to the initial network and to individuals who migrate into the network.

1.2 New injectors

We identified the best-fitting distribution to the observed distribution of ages at injection initiation among the SNAP PWID following the same approach as above, with the lognormal distribution selected as above.

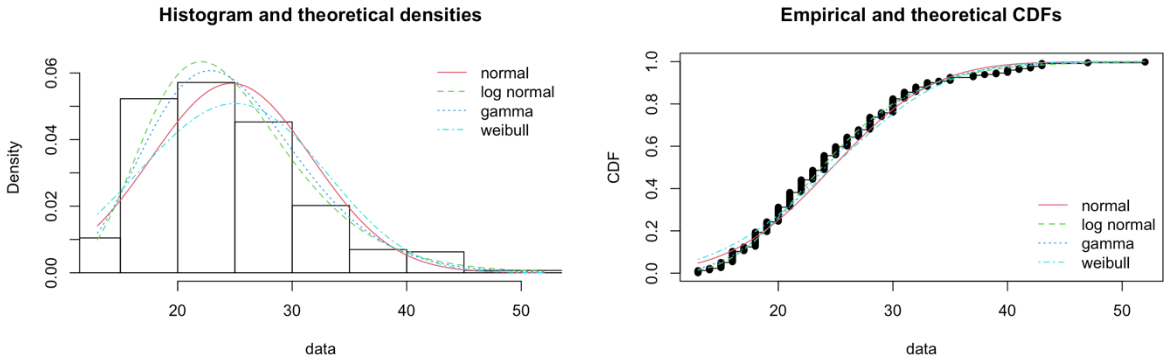

We used the fitted distribution (mean log=3.169, sd log=0.275) truncating at the minimum age of injection initiation = 13 to assign the age distribution for new injectors.

© 2025 Zhu L et al. *JAMA Health Forum*.

1.3 Aging

We updated age for all simulated individuals in the model at yearly intervals.

© 2025 Zhu L et al. *JAMA Health Forum*.

2. Fibrosis distribution

We assigned F0 to individuals who have never been infected. For individuals with current or former infection, we gathered numbers of patients in different fibrosis stages (none/mild, moderate, and advanced/cirrhosis) by birth cohort (born before 1945, 1945-1965, and after 1965) and HCV infection status (currently infected and resolved/cured) from Table 1 in the Klevens et al. study. [9] We converted the numbers of patients to percentages, and mapped none/mild to F0/F1, moderate to F1/F2, and advanced/cirrhosis to F3/F4/decompensated according to matching of FIB-4, Ishak 6, and METAVIR (FIB-4 maps to Ishak 0-1, 2-3, 4-6; Ishak 0-1 maps to METAVIR F0/F1; Ishak 2-3 maps to METAVIR F1/F2; and Ishak 4-6 maps to F3/F4). We implemented 50/50 splits between F0 and F1, F1 and F2, and 50/40/10 split between F3, F4, and decompensated. The table below shows results of these calculations.

Table Fibrosis distribution among individuals with current or former HCV infection

Age	Infection Status	F0	F1	F2	F3	F4	Decomp
65-100	Current	0.0372	0.2665	0.2293	0.2335	0.1868	0.0467
65-100	Resolved/Cured	0.1423	0.4221	0.2798	0.0779	0.0623	0.0156
45-64	Current	0.1465	0.3641	0.2176	0.1359	0.1087	0.0272
45-64	Resolved/Cured	0.3303	0.4693	0.1390	0.0307	0.0245	0.0061
0-44	Current	0.3981	0.4740	0.0759	0.0260	0.0208	0.0052
0-44	Resolved/Cured	0.4713	0.4946	0.0233	0.0054	0.0043	0.0011

© 2025 Zhu L et al. *JAMA Health Forum*.

3. Initiation, cessation, and relapse of injection drug use

3.1 Initiation

We calibrated the initiation rate to make the size of the active injection network stable (around 1000 current PWID) in the status quo (no intervention) scenario. We assumed that each person is HCV naïve at injection drug use initiation.[10]

3.2 Cessation and relapse

We estimated monthly rates of injecting cessation and injecting relapse, and the probability of permanent cessation, from the ALIVE cohort study conducted in Baltimore, MD over 1988 to 2000. [11] The following steps were used:

a. We digitized the time to cessation and relapse curves reported by Shah NG et al. [11] in Figure 1 (Kaplan–Meier of observed time from baseline to first cessation of injection, and time to first relapse to injection following cessation), extracting 25 data points from each curve.
b. We the asymptote of the relapse curve to estimate the probability of permanent cessation, which was approximately 13%.
c. We fitted an exponential function to the digitized data points of the cessation curve with a forced intercept of 100. The exponential function was estimated as $Y = 100 \exp(-0.168 X)$, with an adjusted R-squared of 0.9988. Based on the estimated coefficient, we computed the monthly cessation rate to be $0.168/12 = 0.014$.
d. For the relapse curve, we first converted the Y values to be conditional on ever relapsing (i.e., allowing for the curve to approach an asymptote at 13%): $Z = (Y-13)/(100-13)\times100$. Then we fitted an exponential function using the same method as the cessation curve, and the result was $Z = 100 \exp(-0.454 X)$, with an adjusted R-squared of 0.9589. We computed the monthly relapse rate as $0.454/100\times(100-13)/12 = 0.033$.

PWID who cease injection are characterized as “former PWID” and pause their injection partnerships. When former PWID relapse, they return to the active PWID network and re-acquire previous partners if they remain in the network. PWID who initiate injection are assigned partnerships to preserve network mean degree; partnerships of PWID who die are removed permanently.

© 2025 Zhu L et al. *JAMA Health Forum*.

4. Mortality

4.1 Age dependent base mortality

We used 2019 United States life tables.[12] We downloaded life tables for males (https://ftp.cdc.gov/pub/Health_Statistics/NCHS/Publications/NVSR/70-19/Table02.xlsx) and females (https://ftp.cdc.gov/pub/Health_Statistics/NCHS/Publications/NVSR/70-19/Table02.xlsx) on May 1 2023. We converted mortality probabilities to rates, calculated both-sexes rates using the sex distribution reported for the SNAP PWID sample (59% male), and converted yearly rates back to monthly probabilities. The probability of death at age 100 was set to 1.

4.2 Age-dependent drug-use-related standardized mortality ratio (SMR)

We identified SMRs associated with drug use from the Evans et al.[13] cohort study among individuals accessing pharmacological treatment for opioid dependence, which reported point estimates of 1.8 and 6.1 for SMRs among former and current drug use, respectively, and an overall estimate of 4.5. The study also showed that the SMR decreased by age (Table 3 in the publication), consistent with results of a systematic review [14] and analysis of the ALIVE study. [15] Based on these findings, we estimated age-dependent drug-use-related SMRs using the following calculations.

We calculated the ratios of current or former drug use SMRs to SMRs in the overall cohort with an offset of 1:

$$\text{ratio}_{\text{current}} = \frac{\text{current SMR} - 1}{\text{overall SMR} - 1} = \frac{6.1 - 1}{4.5 - 1} = 1.457$$

$$\text{ratio}_{\text{former}} = \frac{\text{former SMR} - 1}{\text{overall SMR} - 1} = \frac{1.8 - 1}{4.5 - 1} = 0.229$$

We fitted a regression to reported age-specific SMRs in Evans et al., which resulted in the estimated function log(SMR – 1) = 2.6193 – 0.0304 × Age. We used this estimated regression to generate overall SMRs by age, and then used the two ratios calculated in the first step to estimate SMRs by age for current and former injectors.

4.3 Excess mortality due to F4 and decompensation

We calculated excess mortality for compensated and decompensated cirrhosis from results reported in a cohort study by Bruno et al.[16] as follows:

a. From numbers in Figure 1 in the report, the number of liver deaths and annual rate among untreated patients were 60 and 3/100 person-years (py), respectively, implying total person-time of 60/0.03=2000 py; the reported number of liver deaths and annual rate among treated patients without sustained

© 2025 Zhu L et al. *JAMA Health Forum*.

virological response (SVR) were 46 and 2.4/100 py, respectively, implying total person-time is 46/0.024=1917 py.

b. We combined decompensation and HCC in our model as one group representing severe liver disease, so our calculation from this study was also based on this grouping.
c. From Figure 3 in the report, the number of deaths with all combinations of decompensation and HCC from untreated and treated patients without SVR was 30+10+15+47=102, and total person-years was 301+31+251+80=663.
d. Combining numbers in steps a and c, the number of deaths for F4 was 60+46-102=4, person-time was 2000+1917-663=3254, and the annual rate was 4/3254=0.12/100 py, implying a monthly excess mortality rate due to F4 of 0.012/100/12=0.0001.
e. Similarly, the number of deaths for decompensated cirrhosis (plus HCC) was 102/663=15.38/100 py, implying a monthly excess mortality rate due to decompensation (plus HCC) of 0.0128.

4.4 Impact of sustained virologic response (SVR) on excess mortality due to F4 or decompensation

Based on results of an international, multicenter, long-term follow-up study by van der Meer et al. (Table 4 in the report), [17] we multiplied the hazard ratio (HR) for liver-related mortality by SVR and Fibrosis Ishak 6 (corresponding to F4, and we assume similar HR for decompensation) $0.06 \times 4.84 = 0.29$ to derive the HR for liver-related mortality following SVR among F4 and decompensation patients.

© 2025 Zhu L et al. *JAMA Health Forum*.

5. Background health utilities

We used age-specific background health utilities based on population norms measured with the EQ-5D[18] for each individual.

Health utilities for ages below 20 years or ages 90 and older were not reported in the previous study. We set utilities in the 14-19-year age group equal to those in the age group 20-29; and those of age above 89 equal to those reported for ages 80-89.

© 2025 Zhu L et al. *JAMA Health Forum*.

6. Health utilities related to liver conditions and HCV infection

We used values reported in a systematic review and meta-analysis[19] to determine model inputs for utilities related to liver disease and HCV infection status. Calculations are summarized below.

a. The health utility for each individual is determined by age, injection status, and HCV/liver disease status, assuming that each of these three dimensions is independent, and operationalized using a multiplicative model.
b. Utilities for states relating to liver disease were normalized in relation to the utility for sustained virologic response (SVR) to HCV treatment, which we assigned a dimension-specific value of 1.0. We computed utility values for each other state in this dimension as the ratio of the estimated value for that state to the estimated value for SVR, as follows:

   F0-F3 (mild to moderate fibrosis): 0.751/0.786 = 0.96

   F4 (compensated cirrhosis): 0.671/0.786 = 0.85

   Decompensated cirrhosis: 0.602/0.786 = 0.77

c. For changes in utility following treatment, we assumed that those treated in starting states F0-F3 and experiencing SVR would move to a utility value of 1.0, i.e. assumed that effective treatment would reverse disutilities associated with untreated mild to moderate fibrosis. For those treated in starting states of F4 or decompensated cirrhosis, we assumed that SVR would reduce liver disease disutility by a factor of 0.29, which we chose to be the same as the assumed reduction in excess mortality due to F4 and decompensated (as described above, in section 3.4). Based on these assumptions, we calculated utilities for F4 and decompensated after SVR as follows:

   F4: $1 - (1 - 0.85) \times 0.29 = 0.96$

   Decompensated: $1 - (1 - 0.77) \times 0.29 = 0.93$

© 2025 Zhu L et al. *JAMA Health Forum*.

7. Healthcare costs associated with injection drug use

We estimated other healthcare costs (excluding costs of MOUD, detox, etc.) associated with injection drug use and attached them to each individual depending on their age and drug use status. We estimated the costs from the NIDA Clinical Trials Network CTN-0051: Extended-Release Naltrexone vs. Buprenorphine for Opioid Treatment (X:BOT) trial, a multisite, 2-arm, open-label, randomized controlled trial that tested the effectiveness of XR-NTX versus BUP-NX with regard to patient survival, free of opioid relapse, at 24 weeks. [20-22] We contacted the authors (Sean Murphy and Danielle Ryan) to obtain healthcare utilization costs stratified by age, injection drug use status, and treatment. The original results were reported as costs over 6 months and in 2016 dollars. We converted them into monthly costs in 2021 dollars. We presented mean costs across all age groups in the input table in the manuscript. The table below shows the detailed age stratified costs that we used in our simulation.

Table Healthcare utilization costs associated with injection drug use

injection status	age group	cost (6 months, 2016 $)	monthly	2021 $
current	<24 years of age	8731	1455	1687
	25-44	12174	2029	2352
	45-99	10363	1727	2002
former (not on MOUD)	<24 years of age	6380	1063	1233
	25-44	9174	1529	1773
	45-99	7522	1254	1453
former (on MOUD)	<24 years of age	4587	765	886
	25-44	6599	1100	1275
	45-99	5498	916	1062

© 2025 Zhu L et al. *JAMA Health Forum*.

8. Healthcare costs related to HCV and liver conditions

We used costs reported in a retrospective, matched cohort study with a large claims database [23] to calculate background healthcare costs related to HCV and liver conditions. In Table 4 in the cited paper, which summarized the incremental all-cause per patient per year healthcare costs for patients with HCV relative to matched comparison group, we obtained sample sizes, mean inpatient costs, pharmacy costs, and total costs for four HCV and liver condition groups including HCV without liver disease, compensated cirrhosis, decompensated cirrhosis, and HCC. We then adjusted the inpatient costs with a cost-to-charge ratio of 0.329, [24] and subtracted pharmacy costs from total costs to obtain the final background healthcare costs for the four HCV and liver condition groups. To avoid potential measurement error relating to small samples, we collapsed the four categories into two categories, combining HCV without liver disease and compensated cirrhosis into a group spanning F0-F4, and combining decompensated cirrhosis and HCC into a "severe liver disease" category. For each combined category we computed costs as a sample-weighted average of the component category costs. We inflated costs to 2021 US dollars. For patients without HCV or cured, we multiplied costs by a factor calculated from total monthly HCV-related healthcare costs, stratified by SVR attainment status, reported in Table 4 in a matched study from US claims database, [25] which equals 717/1436=0.5. We divided these costs by 12 to obtain monthly costs as our input. The calculations are summarized in the table below.

costs	HCV without liver disease (F0-F3)	Compensated cirrhosis (F4)	Decompensated cirrhosis	HCC
N	26977	1521	4249	959
all-cause healthcare cost total	5870	5330	27845	43671
in-patient cost	810	974	15464	17197
adjusted in-patient cost	266	320	5088	5658
all-cause, adjusted in-patient	5326	4676	17469	32132
pharmacy	2659	3102	0	0
all-case, adjusted in-patient, excluding pharmacy	2667	1574	17469	32132
inflated to 2021	3803	2245	24906	45812
	weighted F0-F4	3720	weighted DC/HCC	28756
	non-HCV	1860		14378

© 2025 Zhu L et al. *JAMA Health Forum*.

Monthly costs input ($):

F0-F4 with HCV: 310

DC with HCV: 2396

F0-F4 without HCV: 155

DC without HCV: 1198

© 2025 Zhu L et al. *JAMA Health Forum*.

eReferences.

1. Boodram B, Hotton AL, Shekhtman L, Gutfraind A, Dahari H. High-risk geographic mobility patterns among young urban and suburban persons who inject drugs and their injection network members. *Journal of Urban Health* 2018; **95**(1): 71-82.

2. Thrash C, Welch-Lazoritz M, Gauthier G, et al. Rural and urban injection drug use in Puerto Rico: Network implications for human immunodeficiency virus and hepatitis C virus infection. *Journal of ethnicity in substance abuse* 2018; **17**(2): 199-222.

3. Hoffmann JP, Su SS, Pach A. Changes in network characteristics and HIV risk behavior among injection drug users. *Drug and alcohol dependence* 1997; **46**(1-2): 41-51.

4. Malekinejad M, McFarland W, Vaudrey J, Raymond HF. Accessing a diverse sample of injection drug users in San Francisco through respondent-driven sampling. *Drug and alcohol dependence* 2011; **118**(2-3): 83-91.

5. Latkin CA, Hua W, Tobin K. Social network correlates of self-reported non-fatal overdose. *Drug and Alcohol Dependence* 2004; **73**(1): 61-7.

6. Latkin C, Mandell W, Oziemkowska M, et al. Using social network analysis to study patterns of drug use among urban drug users at high risk for HIV/AIDS. *Drug and alcohol dependence* 1995; **38**(1): 1-9.

7. Suh T, Mandell W, Latkin C, Kim J. Social network characteristics and injecting HIV-risk behaviors among street injection drug users. *Drug and alcohol dependence* 1997; **47**(2): 137-43.

8. Zelenev A, Li J, Mazhnaya A, Basu S, Altice FL. Hepatitis C virus treatment as prevention in an extended network of people who inject drugs in the USA: a modelling study. *The Lancet Infectious Diseases* 2017.

9. Klevens RM, Canary L, Huang X, et al. The burden of hepatitis C infection–related liver fibrosis in the United States. *Clinical Infectious Diseases* 2016; **63**(8): 1049-55.

10. Fuller CM, Ompad DC, Galea S, Wu Y, Koblin B, Vlahov D. Hepatitis C incidence—a comparison between injection and noninjection drug users in New York City. *Journal of Urban Health* 2004; **81**(1): 20-4.

11. Shah NG, Galai N, Celentano DD, Vlahov D, Strathdee SA. Longitudinal predictors of injection cessation and subsequent relapse among a cohort of injection drug users in Baltimore, MD, 1988–2000. *Drug and alcohol dependence* 2006; **83**(2): 147-56.

12. Arias E, Xu J, Tejada-Vera B, Bastian B. United States life tables, 2019. 2022.

13. Evans E, Li L, Min J, et al. Mortality among individuals accessing pharmacological treatment for opioid dependence in California, 2006–10. *Addiction* 2015; **110**(6): 996-1005.

© 2025 Zhu L et al. *JAMA Health Forum*.

14. Larney S, Tran LT, Leung J, et al. All-cause and cause-specific mortality among people using extramedical opioids: a systematic review and meta-analysis. *JAMA psychiatry* 2020; **77**(5): 493-502.

15. Cepeda JA, Astemborski J, Kirk GD, Celentano DD, Thomas DL, Mehta SH. Rising role of prescription drugs as a portal to injection drug use and associated mortality in Baltimore, Maryland. *PLoS One* 2019; **14**(3): e0213357.

16. Bruno S, Zuin M, Crosignani A, et al. Predicting Mortality Risk in Patients With Compensated HCV-Induced Cirrhosis: A Long-Term Prospective Study. *Official journal of the American College of Gastroenterology| ACG* 2009; **104**(5): 1147-58.

17. van der Meer AJ, Veldt BJ, Feld JJ, et al. Association between sustained virological response and all-cause mortality among patients with chronic hepatitis C and advanced hepatic fibrosis. *Jama* 2012; **308**(24): 2584-93.

18. Hanmer J, Lawrence WF, Anderson JP, Kaplan RM, Fryback DG. Report of nationally representative values for the noninstitutionalized US adult population for 7 health-related quality-of-life scores. *Medical Decision Making* 2006; **26**(4): 391-400.

19. Saeed YA, Phoon A, Bielecki JM, et al. A systematic review and meta-analysis of health utilities in patients with chronic hepatitis C. *Value in Health* 2020; **23**(1): 127-37.

20. Lee JD, Nunes EV, Bailey GL, et al. NIDA Clinical Trials Network CTN-0051, extended-release naltrexone vs. buprenorphine for opioid treatment (X: BOT): study design and rationale. *Contemporary clinical trials* 2016; **50**: 253-64.

21. McCollister KE, Leff JA, Yang X, et al. Cost of pharmacotherapy for opioid use disorders following inpatient detoxification. *The American journal of managed care* 2018; **24**(11): 526.

22. Murphy SM, McCollister KE, Leff JA, et al. Cost-effectiveness of buprenorphine–naloxone versus extended-release naltrexone to prevent opioid relapse. *Annals of internal medicine* 2019; **170**(2): 90-8.

23. McAdam-Marx C, McGarry LJ, Hane CA, Biskupiak J, Deniz B, Brixner DI. All-cause and incremental per patient per year cost associated with chronic hepatitis C virus and associated liver complications in the United States: a managed care perspective. *Journal of Managed Care Pharmacy* 2011; **17**(7): 531-46.

24. Chhatwal J, Ferrante SA, Brass C, et al. Cost-effectiveness of boceprevir in patients previously treated for chronic hepatitis C genotype 1 infection in the United States. *Value in Health* 2013; **16**(6): 973-86.

25. Davis KL, Mitra D, Medjedovic J, Beam C, Rustgi V. Direct economic burden of chronic hepatitis C virus in a United States managed care population. *Journal of clinical gastroenterology* 2011; **45**(2): e17-e24.

© 2025 Zhu L et al. *JAMA Health Forum*.
