# Supplementary material for: Health and Economic Impact of Periodic Hepatitis C Virus Testing Among People Who Inject Drugs
Source: JAMA Health Forum. 2025 Jul 3;6(7):e251870. doi: 10.1001/jamahealthforum.2025.1870 (PMC12232219; doi:10.1001/jamahealthforum.2025.1870)
Supplement: Supplement 2. — Data Sharing Statement [file jamahealthforum-e251870-s002.pdf]

Data Sharing Statement

Zhu. Health and Economic Impact of Periodic Hepatitis C Virus Testing Among People Who Inject Drugs. *JAMA Health Forum*. Published July 03, 2025.
doi:10.1001/jamahealthforum.2025.1870

Data
Data available: No

Additional Information
Explanation for why data not available: We did not collect data in this study.
